# Modeling soil water distribution under drip fertigation in chrysanthemum across soil types using HYDRUS-2D

**DOI:** 10.1038/s41598-026-56155-8

**Published:** 2026-06-02

**Authors:** Atish Sagar, Murtaza Hasan, Dhirendra Kumar Singh, Ali Salem, Dinesh Kumar Vishwakarma, Ozgur Kisi, Pankaj Malkani, Ahmed Elbeltagi, Kadambot H. M. Siddique, Mohamed A. Mattar

**Affiliations:** 1 Division of Agricultural Engineering, ICAR—Indian Agricultural Research Institute, New Delhi, 110012 India; 2 Scientist, Agricultural Engineering, (Krishi Vigyan Kendra, Jalalgarh, Purnea), Bihar Agricultural University, Sabour, Bhagalpur, Bihar 813210 India; 3 Centre for Protected Cultivation Technology, ICAR—Indian Agricultural Research Institute, New Delhi, 110012 India; 4 Civil Engineering Department, Faculty of Engineering, Minia University, Minia, 61111 Egypt; 5 Structural Diagnostics and Analysis Research Group, Faculty of Engineering and Information Technology, University of Pécs, Pécs, 7622 Hungary; 6 Department of Civil Engineering, Graphic Era Deemed to be University, Clement Town, Dehradun, Uttarakhand 248002 India; 7 Department of Civil Engineering, Lübeck University of Applied Sciences, Lübeck, 23562 Germany; 8 Department of Civil Engineering, Ilia State University, Tbilisi, 0162 Georgia; 9 School of Civil, Environmental and Architectural Engineering, Korea University, Seoul, South Korea; 10 Department of Farm Machinery & Power Engineering, Central Agricultural University-College of Agriculture Engineering & Post Harvest Technology, Ranipool, Gangtok, Sikkim 737135 India; 11 Agricultural Engineering Department, Faculty of Agriculture, Mansoura University, Mansoura, 35516 Egypt; 12 The UWA Institute of Agriculture, The University of Western Australia, Perth, WA 6001 Australia; 13 Prince Sultan Bin Abdulaziz International Prize for Water Chair, Prince Sultan Institute for Environmental, Water and Desert Research, King Saud University, P.O. Box 2454, Riyadh 11451, Saudi Arabia

**Keywords:** Moisture content, Emitter discharge, Simulation, Modelling, HYDRUS 2D, Engineering, Environmental sciences, Hydrology

## Abstract

Efficient water management is critical for sustainable chrysanthemum production under protected cultivation was conducted at Indian Agricultural Research Institute (IARI), New Delhi, India. While HYDRUS-based models are widely used for simulating soil water dynamics under drip irrigation, their application in floriculture crops remains limited, particularly for deriving crop-specific irrigation strategies. This study integrates field experimentation (2020–2023) with HYDRUS-2D simulations to evaluate soil water distribution under drip fertigation across multiple soil types. The effects of emitter discharge, irrigation scheduling, and soil hydraulic properties were analyzed to determine optimal root-zone moisture conditions. Results showed that soil moisture remained within the optimal range for up to 48 h after irrigation under appropriate scheduling. Among soil types, moisture retention followed the order: silt ≈ silty clay loam > loam > sandy clay loam > sandy loam. Model validation demonstrated high accuracy (R^2^ = 0.90–0.96; RMSE = 0.011–0.012; Ceff = 0.90–0.92), confirming the reliability of HYDRUS-2D. Simulation results indicated that an emitter discharge of 1.0 lph combined with a 48-hour irrigation interval maintained optimal root-zone moisture while minimizing deep percolation losses. The study highlights the potential of simulation-based approaches for optimizing irrigation design and improving water-use efficiency in greenhouse floriculture systems.

## Introduction

Protected cultivation combined with drip fertigation offers significant potential for year-round production of high-quality vegetables, flowers, and planting material with enhanced resource-use efficiency. By precisely delivering water and nutrients to the root zone, drip fertigation minimizes losses, improves crop performance, and supports sustainable intensification of horticultural systems^1–6^. Among floricultural crops, chrysanthemum (*Chrysanthemum morifolium*) is a prominent nursery-raised cut flower widely favored by floriculturists and entrepreneurs due to its robustness, aesthetic diversity, and high economic returns^6,7^. It ranks third among major cut flowers after the rose and tulip, and is cultivated extensively for both domestic and international markets. Chrysanthemum exhibits remarkable variability in flower color, size, and form, and is characterized by a long-day requirement for vegetative growth and short-day conditions for flowering, enabling its availability throughout the year under controlled environments^6,7^.

Soil moisture is a fundamental variable governing processes across hydrology, agronomy, ecology, meteorology, and climate science^8,9^. As a key component of the hydrological cycle, soil moisture regulates catchment-scale water balance through its interaction with precipitation, evaporation, infiltration, and runoff^10^. Within the crop root zone, soil moisture critically influences plant water availability, root water uptake, land–atmosphere energy exchanges, and hydrological partitioning^11,12^. Maintaining optimal soil moisture conditions is therefore essential for achieving high productivity and efficient water use, particularly under drip fertigation systems where water application is localized and frequent^13–18^. The spatial and temporal distribution of water in soil under drip irrigation depends on multiple interacting factors, including emitter discharge rate, irrigation duration and frequency, soil texture and hydraulic properties, and fertigation scheduling^15,19–25^. In greenhouse cultivation, achieving uniform and adequate moisture availability within the effective root zone remains a major challenge. Inadequate irrigation can induce water stress and reduce yield and quality, whereas excessive irrigation may cause nutrient leaching, oxygen stress, and inefficient water use^26^. A detailed understanding of soil water movement and wetting dynamics is therefore crucial for optimizing irrigation strategies and improving crop performance. Such understanding typically requires a combination of detailed field measurements and robust mathematical modeling approaches^27^.

Direct measurement of soil water distribution in the root zone is often labor-intensive, time-consuming, and spatially limited. Consequently, numerical simulation models have emerged as valuable tools for analyzing soil water dynamics under different irrigation scenarios^28–33^. Models such as HYDRUS-2D employ numerical solutions of the Richards equation to simulate unsaturated water flow, incorporating soil hydraulic properties, boundary conditions, and root water uptake processes. Unlike analytical solutions, which rely on simplifying assumptions and idealized conditions, numerical models can capture real-world heterogeneity in soil properties and irrigation practices, making them particularly suitable for greenhouse and drip irrigation studies^31,34–41^.

The design and management of drip irrigation systems require accurate prediction of wetting patterns around emitters to ensure that the wetted soil volume adequately meets crop water demand without inducing deep percolation or surface runoff. Simulation models assist in determining optimal emitter spacing, discharge rates, and irrigation scheduling by predicting the size and shape of wetted zones, thereby improving root-zone water availability and irrigation efficiency^42–49^. Wetting patterns under drip irrigation can be evaluated either through site-specific experimental studies or through simulation based on mathematical formulations. Most simulation approaches are based on the Richards equation, which describes soil water movement in terms of matric potential or volumetric water content during irrigation events and can be solved using numerical or analytical techniques.

Although several analytical solutions have been developed for simplified forms of the Richards Eqs^50,51^., their applicability is constrained by assumptions such as homogeneous soil conditions and simplified boundary fluxes. As a result, numerical simulation models have been increasingly adopted to study soil water dynamics under surface and subsurface drip irrigation systems^52–54^. Recent studies have further demonstrated the robustness of HYDRUS-based modeling across diverse agroecosystems, including improvements in root water uptake parameterization, accurate simulation of soil moisture and nitrogen transport, and evaluation of management practices such as tillage under varying moisture regimes^53,55^. Previous studies have successfully applied the HYDRUS-2D model to simulate soil moisture dynamics and solute transport under different irrigation systems. For instance, Vishwakarma et al.^15,25^ focused on wetting front dynamics under point-source drip irrigation using Drip-Irriwater, primarily evaluating model performance in predicting soil moisture distribution. While, Sun et al.^32^ investigated water flow and nitrogen transport in paddy fields under traditional flooded irrigation conditions using HYDRUS-2D model, emphasizing model validation against observed data. However, these studies largely remain limited to model calibration and validation for specific field conditions, with less emphasis on root-zone moisture dynamics and irrigation scheduling strategies.

Integrating field experiments with numerical simulation provides a powerful framework for understanding soil moisture dynamics, optimizing water use, and enhancing crop productivity, particularly for water-sensitive crops such as chrysanthemum. Despite its proven scientific reliability, the operational complexity of HYDRUS-2D often limits its direct use by local farmers and policy makers. This highlights the need for simplified and reliable decision-support modules derived from HYDRUS 2D simulations that can translate complex soil–water interactions into practical irrigation strategies. Although HYDRUS based models have been widely applied to simulate soil water dynamics under drip irrigation and their application in protected floriculture systems, particularly chrysanthemum remains limited. Furthermore, most previous studies have focused primarily on model calibration and validation rather than translating simulation outputs into crop-specific irrigation design recommendations. Therefore, the present study extends beyond conventional applications by systematically analyzing wetting pattern dynamics under varying emitter discharge rates and irrigation intervals, integrating field observations with HYDRUS-2D simulations to develop crop-specific irrigation scheduling recommendations for chrysanthemum cultivation under protected conditions across multiple soil textures.

## Materials and methods

### Experimental site, climate and soil

**Fig. 1:**
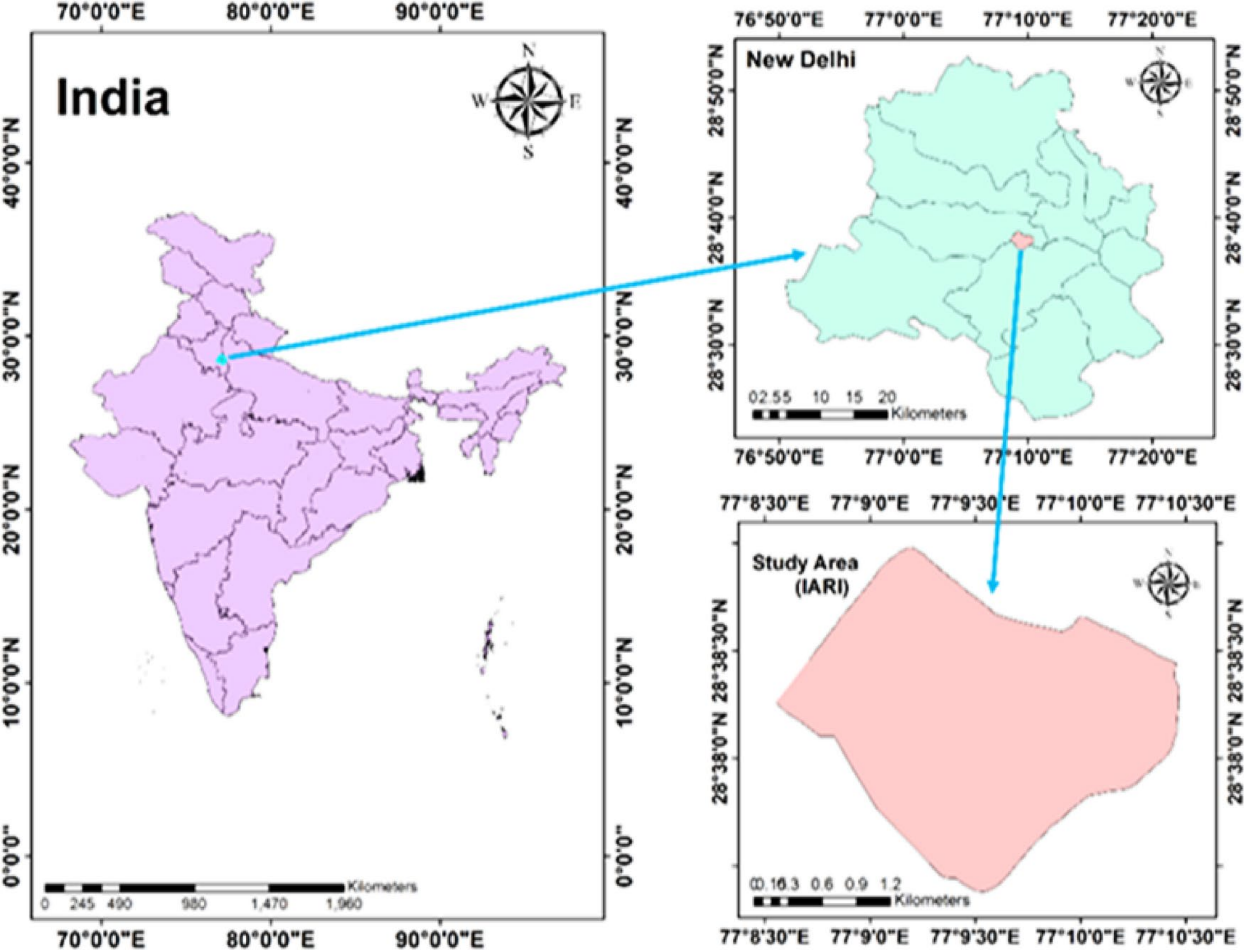
Geographical location of the experimental site at ICAR – Indian Agricultural Research Institute (IARI), New Delhi.

The experiment was conducted at the centre for protected cultivation technology (CPCT) farm of the Indian Agricultural Research Institute (IARI), New Delhi, India. The experimental site is located between 28°37′22″–28°39′00″ N latitude and 77°08′45″–77°10′24″ E longitude, at an elevation of approximately 229 m above mean sea level. The site lies within the 500-ha IARI campus, which is characterized by a gentle natural slope extending from southeast to northwest. The geographical location and layout of the study area within the IARI campus are illustrated in Fig. 1.

The study area experiences a semi-arid subtropical climate typical of the Trans-Gangetic Plains. Summers are extremely hot, with maximum temperatures ranging from 40.8 to 46.5 °C during May–June, while minimum temperatures of 2–5 °C occur in January, occasionally accompanied by frost. The mean annual temperature is 36.7 °C. Average annual rainfall is approximately 710 mm, with 75–80% received during the southwest monsoon (July–September) and light winter showers from December to March. Open pan evaporation peaks in May (13.91 mm/day) and declines to 1.15 mm/day in January. Relative humidity is highest during the monsoon and drops to 20–30% in April–May. Mean wind speed ranges from 3.5 to 6.4 km/h. Meteorological data from the IARI observatory and a Class A pan evaporimeter installed in the greenhouse were used for irrigation scheduling.

The soils are predominantly sandy loam, uniform to a depth of 150 cm, with good porosity (~ 40%) and permeability. Soil samples from 0 to 45 cm depth were analysed for physical properties before transplanting.

### Physical and chemical properties of soil

Soil samples were obtained from successive layers up to a depth of 120 cm and analysed for their physical and chemical properties. The standard methodologies adopted for these analyses are summarized in Table 1. The measured physical properties included particle size distribution, field capacity, bulk density, permanent wilting point, and hydraulic conductivity, while the chemical properties comprised pH, electrical conductivity (EC), nitrogen, phosphorus, potassium, and sulphur contents; these results are presented in Table 2. Soil moisture retention at different suction levels was obtained using a pressure plate apparatus (PPA) to construct the characteristic curve of soil moisture.

**Fig. 2:**
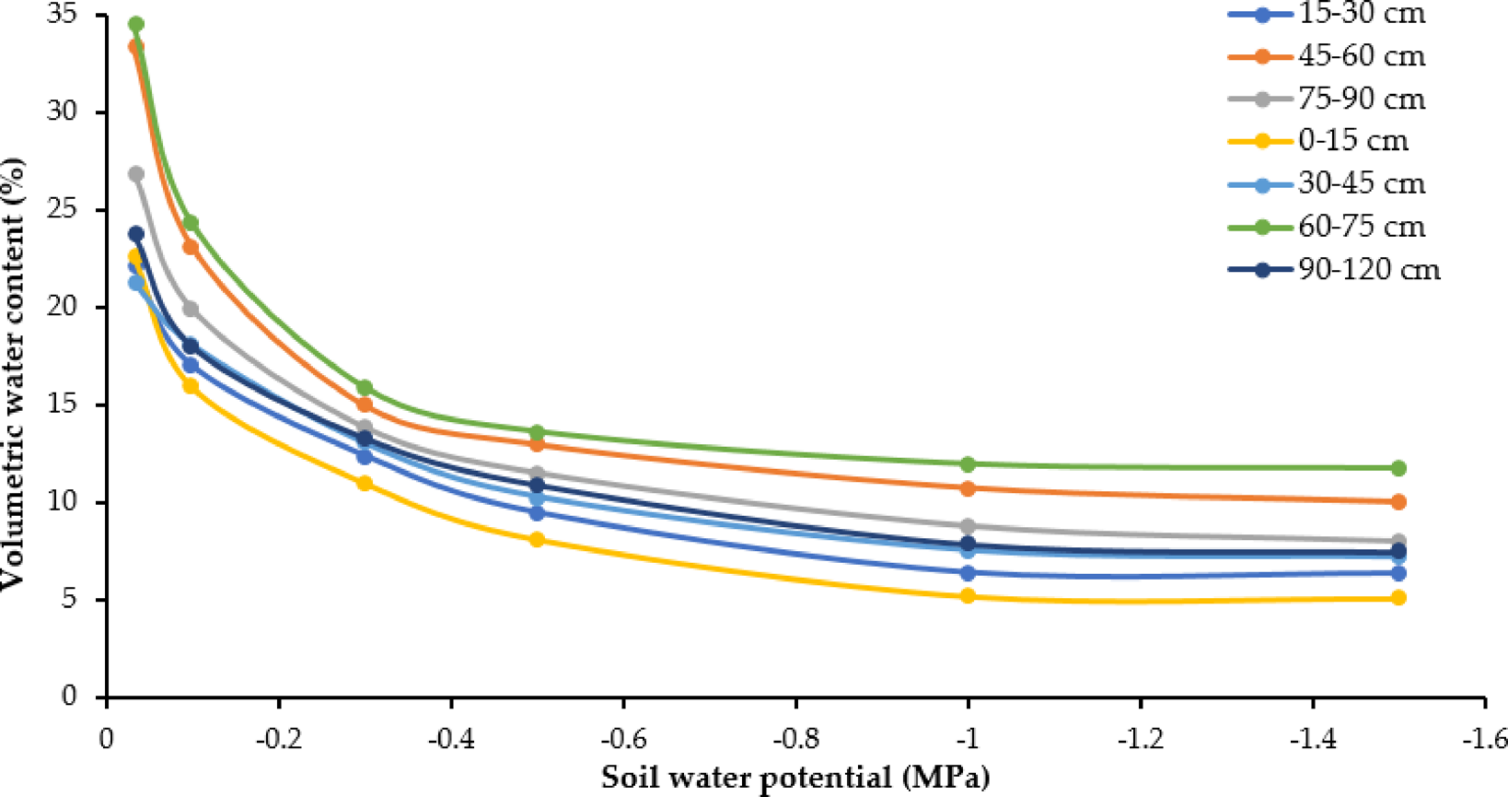
Soil moisture retention curves for the experimental site at seven different depths.

Soil moisture retention at different suction levels was obtained utilizing a PPA to draw the soil moisture retention curve (SMRC). The resulting curves for various soil layers are presented in Fig. 2, which illustrates the moisture retention characteristics of the experimental site across seven distinct soil depths.

**Table 1:** Measured physical and hydraulic characteristics of the sandy loam soil profile before transplanting.

Soil layers (cm)	BD (g/cm^3^)	Particle fraction (%)			Texture (USDA)	OC (%)	FC (%)	PWP (%)
		Clay	Silt	Sand				
0–15	1.52	16	19	65	Sandy Loam	0.22	19.58	6.52
15–30	1.49	14	17	69	Sandy Loam	0.19	17.34	6.45
30–45	1.44	13	21	66	Sandy Loam	0.22	16.65	6.02
45–60	1.68	24	25	51	Sandy Clay Loam	0.19	20.70	7.89
60–75	1.61	25	19	56	Sandy Clay Loam	0.22	21.05	8.41
75–90	1.58	19	23	58	Sandy Loam	0.22	19.05	6.41
90–120	1.59	15	28	57	Sandy Loam	0.17	18.17	6.27

BD = Bulk Density, FC = Field Capacity; PWP = Permanent Wilting Point; OC = Organic Carbon.

**Table 2:** Chemical characteristics of the soil in the experimental field.

Soil layers (cm)	pH	EC (dS/m)	K (cm/h)	Available		
				*N*(Kg/ha)	*P* (Kg/ha)	K (Kg/ha)
0–15	7.2	0.12	1.32	88.76	8.65	139.45
15–30	7.1	0.15	1.49	83.82	7.98	119.90
30–45	7.2	0.12	1.33	88.76	8.65	113.54
45–60	7.1	0.15	1.01	73.85	7.98	91.80
60–75	7.2	0.12	1.05	71.06	8.65	94.65
75–90	7.2	0.12	1.09	78.32	8.65	79.42
90–120	7.2	0.11	1.04	66.98	8.08	92.76

pH = Soil Reaction (potential of Hydrogen); EC = Electrical Conductivity; K = Hydraulic Conductivity; Available N = Available Nitrogen; Available P = Available Phosphorus; and Available K = Available Potassium.

### Experimental treatments and design

The experiment was conducted in a forced-ventilated greenhouse equipped with a low-pressure drip irrigation system. The system consisted of 12-mm lateral lines fitted with emitters delivering 1 lph, with flow regulation achieved through 12-mm control valves installed at the inlet of each lateral. Irrigation water was supplied from a 500-L storage tank positioned 2 m above ground level.

**Fig. 3:**
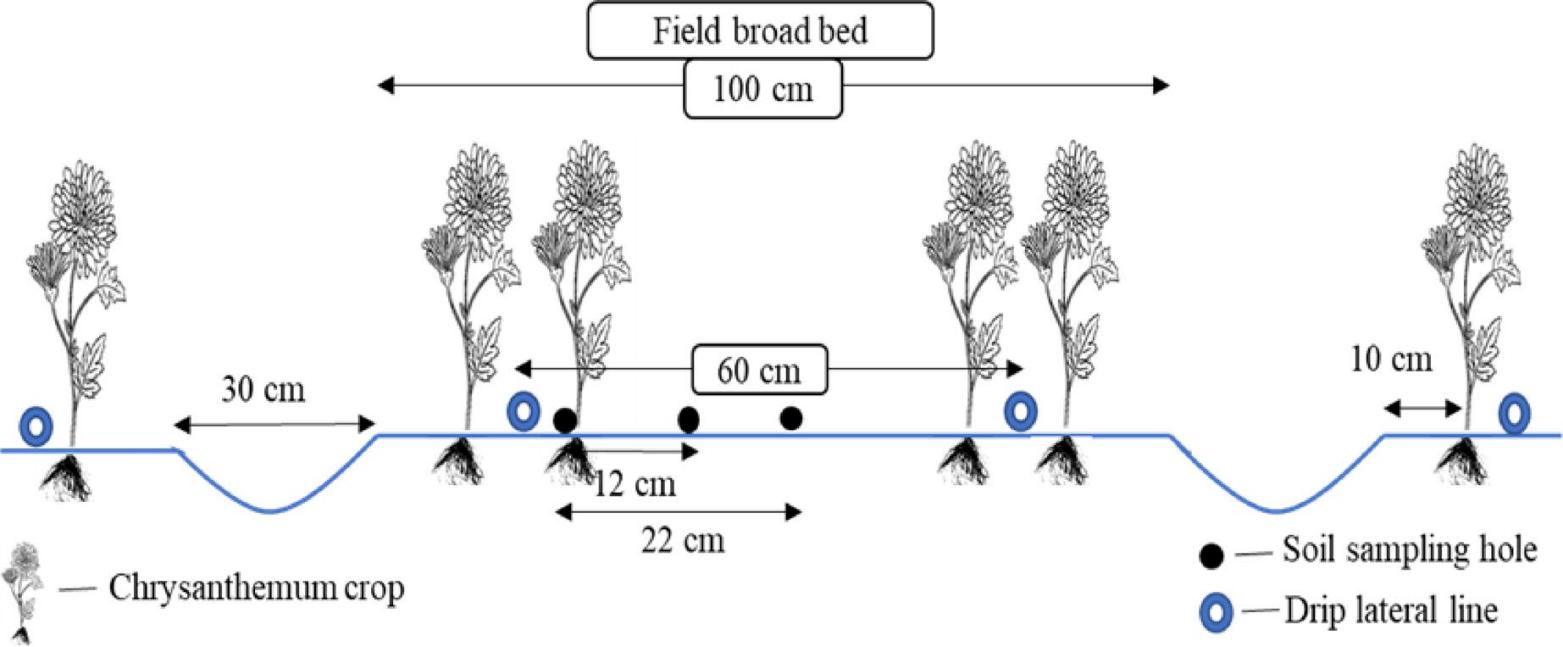
A representation of the planting layout and soil sampling design for chrysanthemum cultivation in the greenhouse.

The study followed a Completely Randomized Design (CRD) with nine treatment combinations and three replications, laid out in homogeneous plots measuring 6.0 × 1.2 m. A Completely Randomized Design (CRD) was adopted given the controlled greenhouse conditions, in which variability in soil properties, temperature, and light distribution was minimal. Under such uniform conditions, CRD is considered appropriate and ensures unbiased comparison among treatments. Each raised bed was fitted with two 12-mm laterals spaced 60 cm apart, with emitters placed at 30-cm intervals. Three irrigation regimes based on pan evaporation—100% (*I*_*1*_), 80% (*I*_*2*_), and 60% (*I*_*3*_)—were factorially combined with three nitrogen levels representing 100% (*F*_*1*_), 80% (*F*_*2*_), and 60% (*F*_*3*_) of the recommended dose of nitrogen (RDN). Raised beds 12 cm high and 120 cm wide were prepared and compacted for uniformity. Owing to the difference between emitter spacing and plant spacing (15 cm), each emitter served two plants. It is important to note that the field experiment was conducted using a fixed emitter discharge of 1.0 lph, whereas variations in emitter discharge (0.5, 1.0, and 2.0 lph) were introduced only in simulation scenarios to evaluate their effects on soil water distribution. The drip layout and planting geometry are presented in Fig. 3.

### Irrigation scheduling

Irrigation scheduling involves determining the amount, timing, interval, and duration of irrigation. For chrysanthemum, the crop water requirement was determined utilizing the pan evaporation (*E*_*pan*_) approach, where the five-year average daily values of *E*_*pan*_ were multiplied by corresponding pan and crop coefficients to calculate the daily water needs. Effective rainfall was negligible during the study, and thus not considered. Differences in estimated water requirements between the first and second years reflected variability in the preceding five-year *E*_*pan*_ records. Irrigation was applied on alternate days to meet the cumulative two-day crop water demand, resulting in variable irrigation depths depending on evaporative demand. On average, three irrigation events per week were scheduled.

Daily irrigation water requirement (*IWR*) was estimated by using the Eq. 1. The net applied water volume per irrigation (*NIR*) was estimated for greenhouse chrysanthemum from *IWR*, irrigation interval (*P*), effective rainfall $\:\left({R}_{eff\:}\right)$ and the irrigation efficiency (*η* = 90%) (Eq. 2). The irrigation duration (*t*) for each cycle was determined from net applied irrigation volume and dripper discharge (q) (Eq. 3).

1 $$\:IWR=\:\frac{{ET}_{o}\times\:{K}_{c}\times\:w\times\:d\times\:P}{\eta\:}$$

2 $$\:NIR=IWR-{R}_{eff\:}$$

3 $$\:t=\:\frac{NIR}{q}$$

where, $\:{ET}_{o}$ = Reference evapotranspiration (mm/day), *K*_*c*_ = Crop coefficient (dimensionless), *w* = Width of the cropped area or plot (m), *d* = Depth of root zone or effective soil depth considered (m), *P* = Irrigation interval (days), *η* = Irrigation efficiency (%), *IWR* = Irrigation water requirement (mm, depending on unit system), $\:{R}_{eff\:}$= Effective rainfall (mm), *NIR* = Net irrigation requirement (mm), *q* = Dripper discharge rate (lph), *t* = Irrigation duration per cycle (hours).

### Field observations

The procedures applied for field observations are detailed in the following section, with the observation schedule summarized in Table 3. The content of soil water at depths of 0–15, 15–30, and 30–45 cm was obtained utilizing the gravimetric method. For this, soil samples were collected with a screw auger in aluminum cans, weighed immediately, and then oven-dried at 105 °C for at least 24 h. In absence of yield measurements, soil moisture availability and depletion trends were used as proxies to assess irrigation efficiency. The dried samples were reweighed, and soil moisture content on a mass basis was computed by Eq. 4:

4 $$\:SM\:\left(\%\right)=\frac{{M}_{w}-\:{M}_{d}}{{M}_{d}}\:\:\times\:100$$

where, *SM* = soil moisture (%), $\:{M}_{w}$ = soil sample weight before drying and $\:{M}_{d}$ = the sample weight after drying.

**Table 3:** Schedule of different soil and plant observations during the experimental period.

a) Soil parameters		
Parameters	Observation time	
Bulk Density	Before Planting i.e. at initial stage	
Soil Moisture	Before irrigation, (2 h, 4 h, 24 h, 48 h after 1st irrigation), (2 h, 24 and 48 h after next irrigation), 1-month, flowering stage and harvesting stage.	
FC; PWP & Texture	Before Planting i.e. at initial stage	
K_s_	Before Planting i.e. at initial stage	

b) Plant parameters		
Parameters	Observation period	Frequency of observation
LAI	15, 30, 45 and 60 DAT	4 times
Root sampling	Flowering Stage	one

### Model description

The HYDRUS-2D model is a Microsoft Windows–based software package designed to simulate the temporal dynamics of soil water distribution and root water uptake^56–58^. It uses the finite element method to numerically solve Richards’ equation, which governs soil water flow and is expressed as follows:

5 $$\:\frac{\partial\:\theta\:}{\partial\:t}=\frac{\partial\:}{\partial\:x}\left[D\left(\theta\:\right)\frac{\partial\:\theta\:}{\partial\:x}\right]+\frac{\partial\:}{\partial\:z}\left[D\left(\theta\:\right)\frac{\partial\:\theta\:}{\partial\:z}\right]\pm\:{S}_{r}$$

where, $\:\theta\:$ denotes the water content, $\:D\left(\theta\:\right)$ represents the hydraulic diffusivity as a function of $\:\theta\:$, $\:{S}_{r}$ is the sink term, indicating the volume of water removed per unit time from a unit volume of soil due to plant water uptake.

In other words, *S* represents root water uptake, which can be estimated utilizing the Feddes root water uptake (RWU) model. Further information about the model is provided in the HYDRUS-2D manual^59^.

#### Governing water flow equation

The governing flow equation in HYDRUS-2D is based on two-dimensional, isothermal Darcian flow through a variably saturated, rigid porous medium. It is assumed that the influence of the gaseous phase on liquid water movement is negligible, such that water flow is primarily controlled by soil matric and gravitational potentials. Accordingly, the model employs a modified form of Richards’ equation, expressed as follows:

6 $$\:\frac{\partial\:\theta\:}{\partial\:t}=\:\frac{\partial\:}{\partial\:{x}_{i}}\left[K\left(\theta\:\right)\:{K}_{ij}^{A}\left(\:\frac{\partial\:h}{\partial\:{x}_{j}}+\:{\delta\:}_{iz}\right)\right]-S$$

where, *θ* = volumetric water content [L^3^/L^3^], *h* = pressure head [L], $\:\frac{\partial\:h}{\partial\:{x}_{j}}$ = matric potential gradient, $\:{\delta\:}_{iz}$ = gravitational gradient, $\:K\left(\theta\:\right)$ → unsaturated hydraulic conductivity [L/T], *S* = sink term (root water uptake) [1/T], *x*_*i*_ [*i* = 1, 2] = spatial coordinates [L], *t* = time [T], $\:{K}_{ij}^{A}$ = components of a dimensionless anisotropy tensor.

If isotropic:

7 $$\:\frac{\partial\:\theta\:}{\partial\:t}=\:\nabla\:\cdot\:\left[K\left(\theta\:\right)\:\left(\:\nabla\:h+\nabla\:z\right)\right]-S$$

Unsaturated hydraulic conductivity function $\:K\left(\theta\:\right)$ given as:

8 $$\:K\left(h,x,z\right)=\:{K}_{s}\left(x,z\right){\:K}_{r}\left(h\right)$$

where, $\:{K}_{r}$ = the relative hydraulic conductivity and $\:{K}_{s}$ the saturated hydraulic conductivity [L/T].

The anisotropy tensor $\:{K}_{ij}^{A}$ in (Eq. 6) is utilized to account for an anisotropic medium.

For axisymmetric flow, commonly used to represent flow around point sources or emitters, Richards’ equation in cylindrical coordinates (three-dimensional) is expressed Eq. (9)^60–62^:

9 $$\:\frac{\partial\:\theta\:}{\partial\:t}=\:\frac{1}{r}\frac{\partial\:}{\partial\:r}\left[rK\left(h\right)\frac{\partial\:h}{\partial\:r}\right]+\frac{\partial\:}{\partial\:z}\left[K\left(h\right)\left(\frac{\partial\:h}{\partial\:z}+1\right)\right]-S$$

where, *θ* represents the volumetric water content (*L*^*3*^*/L*^*3*^), $\:h$ is pressure head (*L*), *t* is time (*T*), *r* is radial coordinate (*L*), *z* is vertical coordinate (*L*), taken positive upward, and the term + 1 represents the gravitational potential gradient in the vertical direction. and $\:K\left(h\right)$ is unsaturated hydraulic conductivity (*L/T*).

#### System geometry

**Fig. 4:**
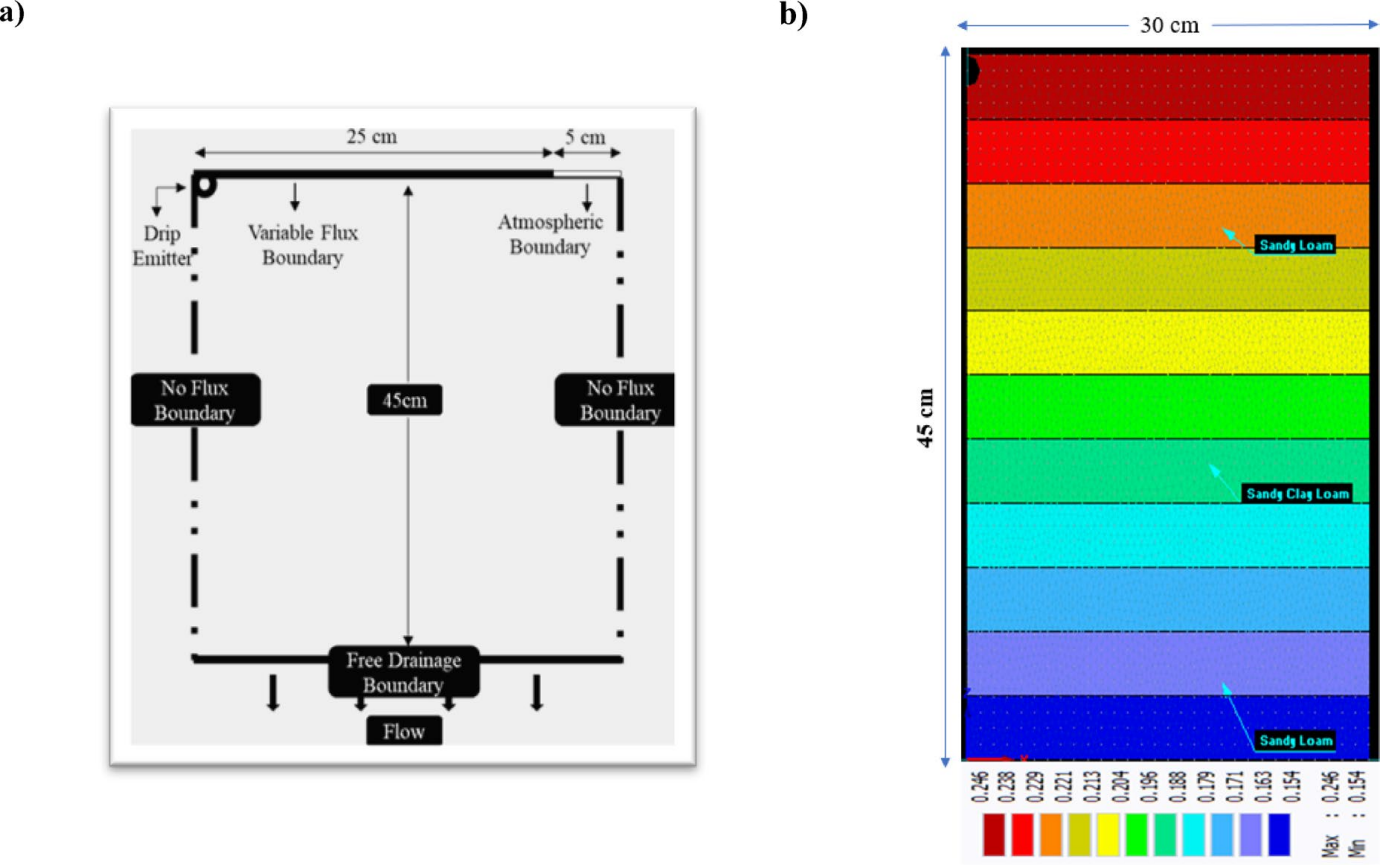
(**a**) Conceptual diagram of simulated area and (**b**) Initial condition describing the soil water distribution content.

The simulations were conducted for a soil profile extending to a depth of *z* = 45 cm and a radial distance of *r* = 30 cm, with a trickle emitter positioned at the soil surface. The flux radius was assumed equal to the wetted radius, with the emitter located at the center of the profile. Based on this flux radius, the surface area available for irrigation without inducing ponding was determined, and the corresponding flux per unit area for a single emitter was subsequently estimated. A conceptual schematic of the simulated domain and the applied boundary conditions is illustrated in Fig. 4a. No-flux conditions were imposed along the lateral boundaries, while the bottom boundary was treated as a free drainage boundary. At the soil surface, a variable flux boundary was prescribed within a 25-cm radius around the emitter, and the remaining 5-cm radial distance was subjected to atmospheric boundary conditions. To consider the spatial heterogeneity in soil physical properties, the soil profile was stratified into four distinct layers. The entire simulated domain was discretized into finite elements with an average dimension of 0.50 cm × 2.06 cm, as illustrated in Fig. 4b.

#### Initial and boundary conditions

The initial distribution of soil water content within the flow domain was assigned according to values measured in the experimental field. A representative illustration of the initial water content profile is shown in Fig. 4b. To evaluate the effects of emitter discharge rate, soil hydraulic properties, and irrigation frequency on wetting patterns, a time-dependent flux boundary condition was imposed at the soil surface within a 25-cm radius from the emitter. This approach allowed simulation of both irrigation and non-irrigation periods, as well as temporal variations in irrigation duration over the crop growth cycle. Since the groundwater table was located well below the domain of interest, a free drainage boundary condition was applied at the lower boundary of the soil profile. Along the lateral boundaries, no-flux conditions were prescribed, assuming negligible water movement across these sides. In HYDRUS-2D, such no-flux boundaries are specified for impermeable interfaces, where the water flux normal to the boundary is considered zero.

#### Simulation of water and root water uptake and model calibration

**Fig. 5:**
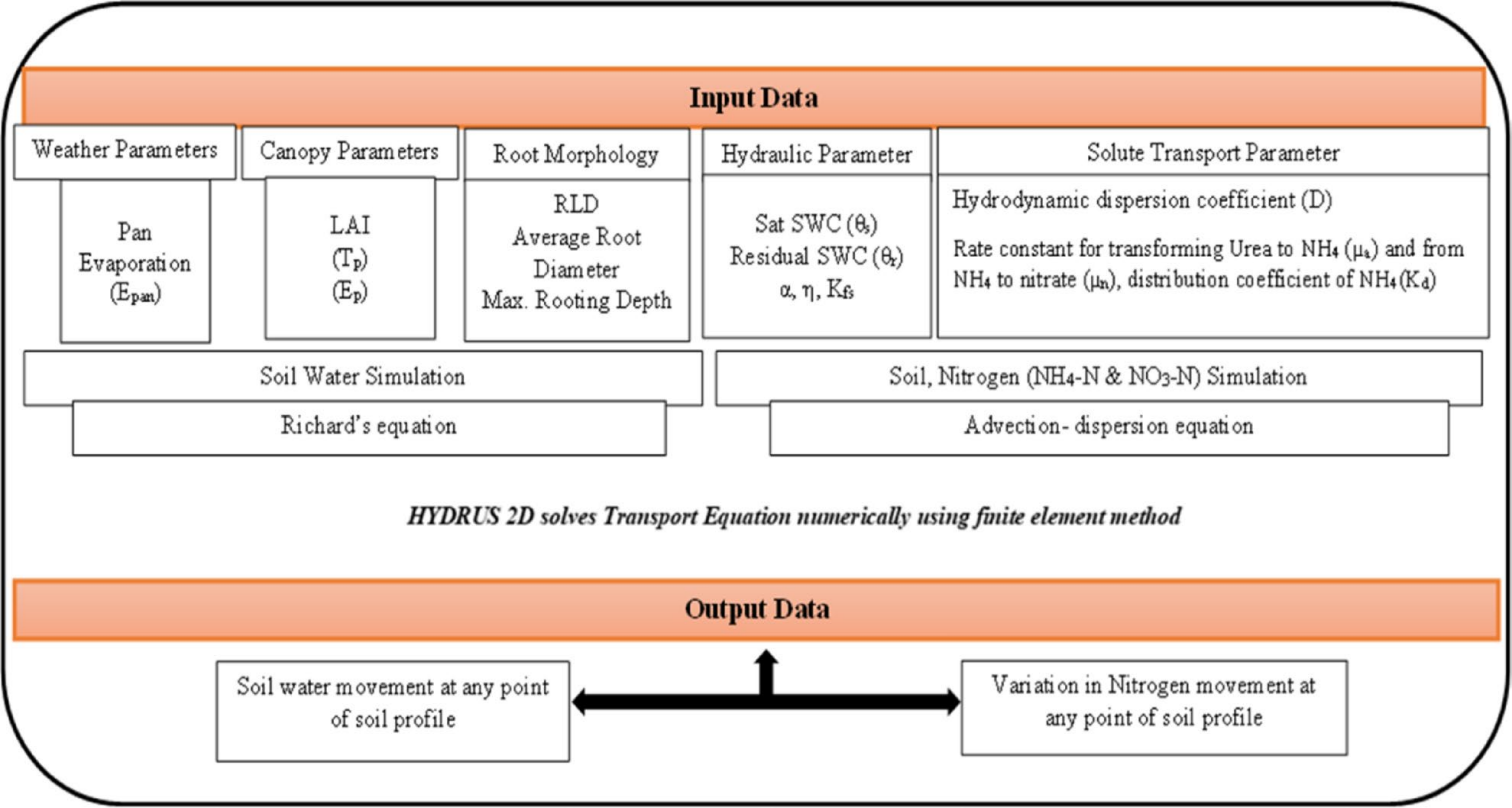
Required input and output parameters in the HYDRUS-2D model for simulating soil water and nitrogen transport in the soil profile.

Model calibration involved iterative adjustment of soil hydraulic parameters using HYDRUS-2D until simulated soil water content matched field observations (Fig. 5). The model calibration steps are detailed below:

- **Soil hydraulic properties and hydraulic sub model used for calibration**.

Parameter optimization in HYDRUS-2D was performed using the Marquardt–Levenberg algorithm, which minimizes the difference between observed and simulated soil moisture values. The stopping criteria were defined based on convergence tolerance limits and minimization of the objective function (RMSE), ensuring stable and reliable parameter estimation. Hydraulic parameters for soil textures other than the experimental sandy loam were obtained using the Rosetta pedotransfer function based on soil texture and bulk density inputs and were supplemented with values reported in the literature. This approach enabled comparative simulation across multiple soil types without direct field measurement. Soil moisture behavior is commonly described using the Brooks–Corey and van Genuchten models. Given that soils under drip irrigation remain near saturation during the crop season, the van Genuchten–Mualem model. without hysteresis was adopted in this study to estimate the parameters of the soil moisture characteristic curve and the unsaturated hydraulic conductivity *K(θ)*, essential for simulating the daily soil water content (WC) profile. The effective saturation ($\:{S}_{e}$) used in the van Genuchten–Mualem model:

10 $$\:{S}_{e}=\:\frac{\theta\:-{\theta\:}_{r}}{{\theta\:}_{s}-{\theta\:}_{r}}$$

11 $$\:{S}_{e}={\left(\frac{1}{1+{\left(\alpha\:\psi\:\right)}^{n}}\right)}^{m}$$

where, $\:\theta\:$ = volumetric WC (cm^3^/cm^3^), $\:{\theta\:}_{r}$ = residual WC (cm^3^/cm^3^), $\:{\theta\:}_{s}$ = saturated WC (cm^3^/cm^3^), $\:\psi\:$ = soil water pressure head (cm), $\:\alpha\:$ = inverse of air-entry suction (1/cm), *n* = pore-size distribution parameter (dimensionless) and $\:m=1-\frac{1}{n}$.

Equation 10 defines effective saturation as a normalized measure of WC between residual and saturation, while the Eq. 11 expresses $\:{S}_{e}$ as a function of pressure head, linking soil water retention with hydraulic properties. Mualem–van Genuchten model for unsaturated hydraulic conductivity shown in Eq. 12:

12 $$\:K\left(\theta\:\right)={K}_{s}{ \cdot \:S}_{e}^{l}{\left[1-{\left(1-{{S}_{e}}^{\raisebox{1ex}{$l$}\!\left/\:\!\raisebox{-1ex}{$m$}\right.}\right)}^{m}\right]}^{2}$$

where, $\:K\left(\theta\:\right)$ = unsaturated hydraulic conductivity (cm/h or cm/day), $\:{K}_{s}$ = saturated hydraulic conductivity (cm/h or cm/day), $\:{S}_{e}$ = effective saturation (dimensionless), *l* = pore connectivity/tortuosity parameter (dimensionless, usually ~ 0.5), *m* = van Genuchten shape parameter, related to *n* as $\:m=1-\frac{1}{n}$.

Temporal discretization was implemented using finer time steps (hourly) during irrigation events and coarser time steps (daily) during redistribution periods to ensure numerical stability and computational efficiency. For modeling the K–θ relationship using the van Genuchten hydraulic model, five input parameters are required (K_s_, θ_r_, θ_s_, α, and n). In this study, α and n were obtained using the Rosetta Lite submodule^63^, which predicts soil hydraulic properties through an artificial neural network based on inputs such as the distribution of particle sizes (sand, silt, clay), bulk density, and soil water content at 0.33 bar. Whereas, measured values of field saturated hydraulic conductivity ($\:{K}_{s}$) at 0–15, 15–30 and 30-45-cm layer and residual soil water content ($\:{\theta\:}_{r}$) (SWC at 15 bar) and saturated soil water content of the undisturbed cores obtained from the above-mentioned three layers under treatment T_2_.

b) **Root water uptake (RWU) model**.

Root water uptake (RWU), represented as a sink term in Richards’ equation, was simulated utilizing the Feddes model (Feddes et al., 1974). The uptake function is expressed as:

13 $$\:S\left(\psi\:,x,z\right)={\upalpha\:}\left(\psi\:,x,z\right)\times\:b\left(x,z\right)\times\:TP\times\:L$$

where, $\:S\left(\psi\:,x,z\right)$ = actual root water uptake rate (cm^3^/cm^3^/d), $\:{\upalpha\:}\left(\psi\:,x,z\right)$ = water stress response function, dependent on soil water pressure head ψ and position (dimensionless), $\:b\left(x,z\right)$ = normalized root distribution function in space (dimensionless), *TP* = transpiration rate under potential conditions (cm/d), *L* = root length density (cm/cm^3^).

Root water uptake was simulated using the macroscopic Feddes model, which distributes water uptake based on root density and soil water pressure head. While this approach is computationally efficient and widely applied, it simplifies complex root–soil interactions and does not explicitly represent root architecture or dynamic growth processes. Root distribution parameters were measured at the flowering stage, corresponding to peak root development, and were assumed to remain constant during the simulation period. This represents a simplification of actual root growth dynamics and may introduce uncertainty when extrapolating results across the entire crop cycle. Root water uptake was simulated using the macroscopic Feddes model, which provides a simplified representation of soil–plant water interactions and does not explicitly account for dynamic root growth or architecture.

c) **Water stress response function** $\:\boldsymbol{\upalpha\:}\left(\boldsymbol{\psi\:},\boldsymbol{x},\boldsymbol{z}\right)$.

According to the HYDRUS-2D database, the value of the water stress response function (α) was set to 1 when the soil water pressure head ($\:\psi\:$) ranged between − 100 and − 1000 cm under maximum daily potential transpiration, and between − 100 and − 3000 cm under minimum daily potential transpiration during the simulation period. Beyond these ranges, α decreased linearly as *ψ* declined from − 1000/–3000 cm to − 15,000 cm, representing the wilting point.

d) **Normalized water uptake distribution function** $\:\boldsymbol{b}\left(\boldsymbol{x},\boldsymbol{z}\right)$.

This function characterizes the spatial distribution of potential water extraction within the root zone. The parameters required for determining $\:b\left(x,z\right)$ included maximum rooting depth, depth of maximum root intensity, maximum rooting radius (i.e., the horizontal extent of roots from the plant base), and the radius of maximum horizontal root intensity (average root radius in cm). These root parameters were quantified through root sampling conducted primarily at the flowering stage of chrysanthemum growth, when root development was near its peak.

e) **Time variable atmospheric boundary conditions**.

The HYDRUS-2D model requires daily inputs of rainfall, irrigation, potential soil evaporation (*EP*), and potential plant transpiration (*TP*) to determine the upper atmospheric boundary condition.

f) **Computation of potential evaporation rate (*EP*) and potential transpiration rate (*TP*)**.

The first step is the estimation of daily ET_o_ from the class A pan evaporimeter installed inside the greenhouse. The second step was to compute ET_c_ which was obtained by multiplying ET_o_ with appropriate crop coefficient (K_c_) (which varies with crop type and crop stage). The value of K_c_ for chrysanthemum was acquired from the FAO-56 manual of the same family crop since the value of K_c_ is not available in the manual. For partitioning ET_c_ into EP and TP, the following formula (Ritchie, 1972) were utilized:

14 $$\:TP={ET}_{c}\times\:{CF}_{Surface}$$

15 $$\:EP={ET}_{c}\times\:\left(1-{CF}_{Surface}\right)$$

16 $$\:{CF}_{Surface}=1-{exp}\left(-0.46\times\:LAI\right)$$

where $\:{CF}_{Surface}$ = the Surface Cover Fraction obtained from the Leaf Area Index (LAI). Evapotranspiration, evaporation, and transpiration are expressed in mm. Daily LAI values were derived from the regression equation generated between the observed LAI and crop period.

g) **Calibration and validation period**.

Calibration and validation period for daily SWC prediction was done for the flowering stage of the crop growth. The full crop growth period was not considered for simulation because the model requires root growth parameters, and the root growth module for RWU estimation is static, preventing the inclusion of temporal root expansion. Consequently, shorter calibration periods were adopted, using root growth parameters measured at the midpoint of each period to represent the entire duration. The assumption of static root distribution represents a simplification of actual root growth dynamics. Although commonly used in HYDRUS simulations, this assumption may introduce uncertainty when extrapolating results over the entire crop growth period. Treatment T₂ was selected for calibration as it represented optimal irrigation conditions, characterized by moderate water application and uniform wetting patterns within the root zone. This ensured that the calibrated hydraulic parameters were derived under representative soil moisture conditions, avoiding bias associated with either deficit or excessive irrigation. A similar approach is commonly adopted in HYDRUS-based studies where calibration is performed under representative treatment conditions and subsequently extended to broader simulation scenarios. Calibration and validation were performed using independent datasets. The calibration process utilized selected soil moisture observations for inverse modeling, while validation was conducted using separate datasets collected at different spatial locations (at emitter, 12 cm, and 22 cm from emitter) and time intervals. This ensured that model performance was evaluated independently of the calibration dataset.

In this study, the 65–91 DAT window was chosen as it coincides with the flowering stage, during which root growth peaks and a static root distribution can be assumed. Although the number of observations used for inverse modeling was limited, the dataset captured key temporal and spatial variations in soil moisture across depths and irrigation intervals. HYDRUS-2D inverse modeling has been shown to provide stable parameter estimation when representative datasets covering the range of system dynamics are used. After calibration and validation of the model, simulation was done for entire crop growth period.

h) **Inverse modelling procedure for optimisation of hydraulic parameters needed for model calibration**.

Among the inputs needed for modeling *K-θ* relation viz. *K*_*s*_, *θ*_*r*_, *θ*_*s*_, *α* and *n* of the Van Genucheten hydraulic model, only *K*_*s*_, *α* and *n* were selected for optimization. The steps of optimization are:

- Arbitrary minimum and maximum values were selected for *K*_*s*_, *α* and *n*.
- Daily time variable boundary conditions i.e. information on daily rainfall, EP and TP were mentioned along with the information on root distribution parameters.
- Initial condition for SWC was given as value of SWC measured at the time of transplanting i.e. 0 DAT.
- Nine observed SWC data points from different depths during the flowering stage were used as the training dataset for treatment T₂ to optimize model parameters through inverse modeling.”
- The inverse modeling procedure utilized measured *K*_s_, *θr*, and *θ*_s_, along with predicted α and n values from the Rosetta Lite submodule, to estimate the optimized calibration parameters (*K*_s_, *α*, and *n*) for subsequent model validation. Parameter optimization is widely employed in science and engineering to enhance model predictive accuracy by minimizing the discrepancy between observed and simulated outputs, thereby enabling more reliable and precise simulations^64–67^. Temporal discretization was implemented using variable time steps, with hourly intervals during irrigation events to capture rapid soil moisture changes and daily intervals during redistribution periods to ensure computational efficiency while maintaining numerical accuracy.

**Fig. 6:**
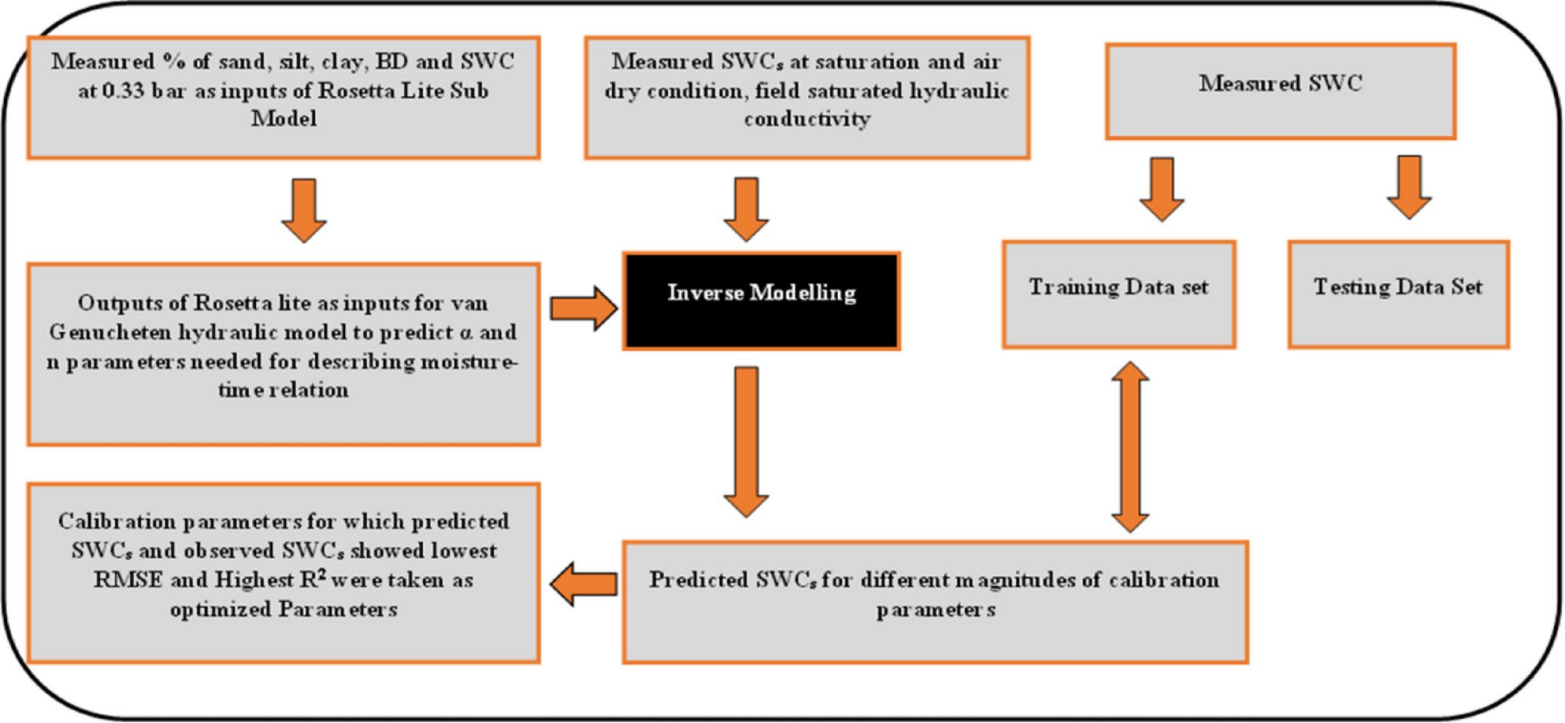
Flowchart of the model calibration procedure using inverse modeling to optimize soil hydraulic parameters in HYDRUS-2D.

**Fig. 7:**
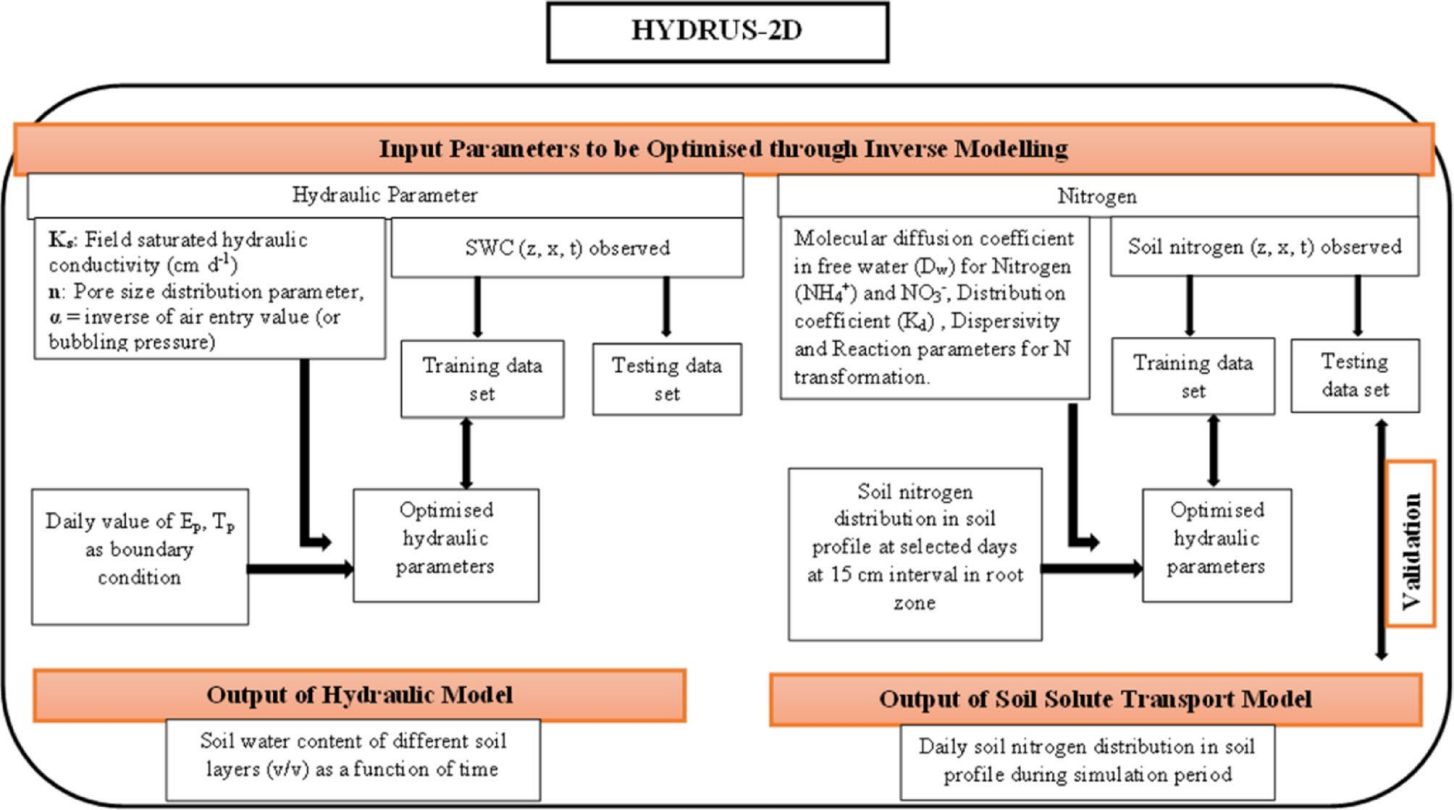
Flowchart showing inverse modeling technique used in HYDRUS 2D for optimisation of hydraulic parameters needed for simulation of temporal distribution of soil water content (SWC) in soil profile.

**Fig. 8:**
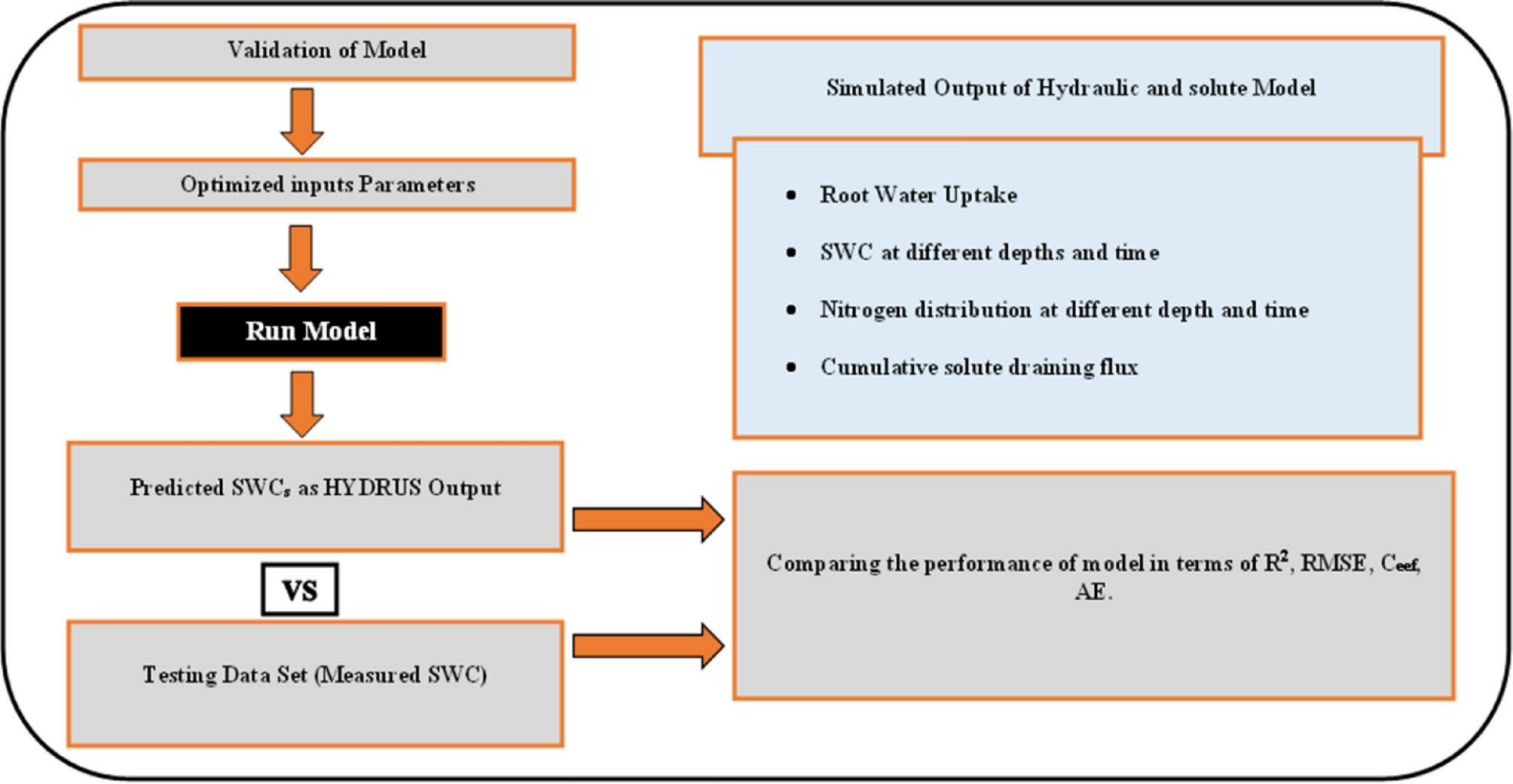
Workflow for model validation and assessment of simulation outputs in HYDRUS-2D.

Figures 6 and 7 describe the inverse modelling technique used in HYDRUS 2D for optimisation of hydraulic parameters needed for simulation of the temporal distribution of soil water content (SWC) in the soil profile whereas, Fig. 8 shows the steps for validation of the model and the output parameters of the simulation.

### Model validation

The performance of HYDRUS-2D was evaluated using three statistical indicators: Average Error (*AE*), Root Mean Square Error (*RMSE*), and the Coefficient of Efficiency (*C*_*eff*_), which were computed using Eqs. 17–19. Absolute error reflects model bias, with a value of zero indicating perfect agreement between observed and simulated values. The root mean square error represents the average magnitude of prediction errors and should be minimized to ensure higher simulation accuracy. The efficiency coefficient evaluates model performance relative to the variability in observed data, where a value of 1.0 denotes perfect prediction, a value of 0 indicates no improvement over using the mean of observations, and negative values imply poor predictive capability^68–71^.

**Table:** 

Equation	Range	Ideal value	Reference	
$\:AE={\sum\:}_{i=0}^{N}\frac{\left({S}_{i}-\:{O}_{i}\right)}{N}\:\:\:\:\:\:\:\:\:\:$	–∞ to +∞	0	Willmott^72^	(17)
$\:RMSE=\sqrt{{\sum\:}_{i=0}^{N}\frac{{\left({S}_{i}-\:{O}_{i}\right)}^{2}}{N}}$	0 to +∞	As small as possible (0 for perfect fit)	Willmott and Matsuura^73^	(18)
$\:{C}_{eff}=1-\frac{\sum\:_{i=1}^{N}\:{\left({S}_{i}-\:{O}_{i}\right)}^{2}}{\sum\:_{i=1}^{N}\:{\left({O}_{i}-\:\stackrel{-}{O}\right)}^{2}}$	–∞ to 1	1	Nash and Sutcliffe^74^	(19)

where, $\:{S}_{i}$ = simulated value, $\:{O}_{i}$ = observed value, N = number of observations and $\:\stackrel{-}{O}$ = mean of observed values.

## Results and discussion

### Spatial and temporal distribution of water during growing period

**Fig. 9:**
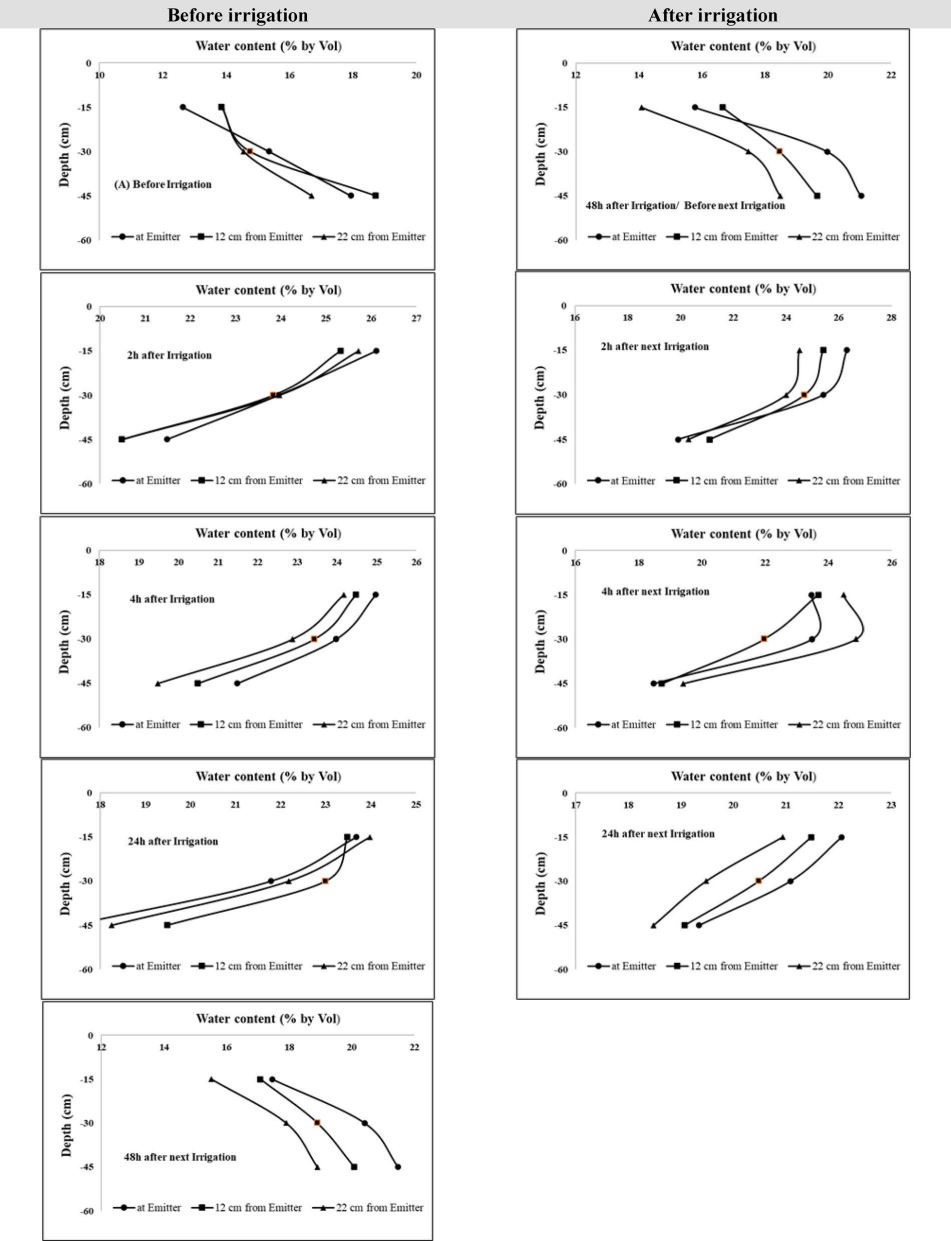
Average water distribution pattern for treatments *T*_*1*_, *T*_*2*_ and *T*_*3*_ two months after transplanting.

**Fig. 10:**
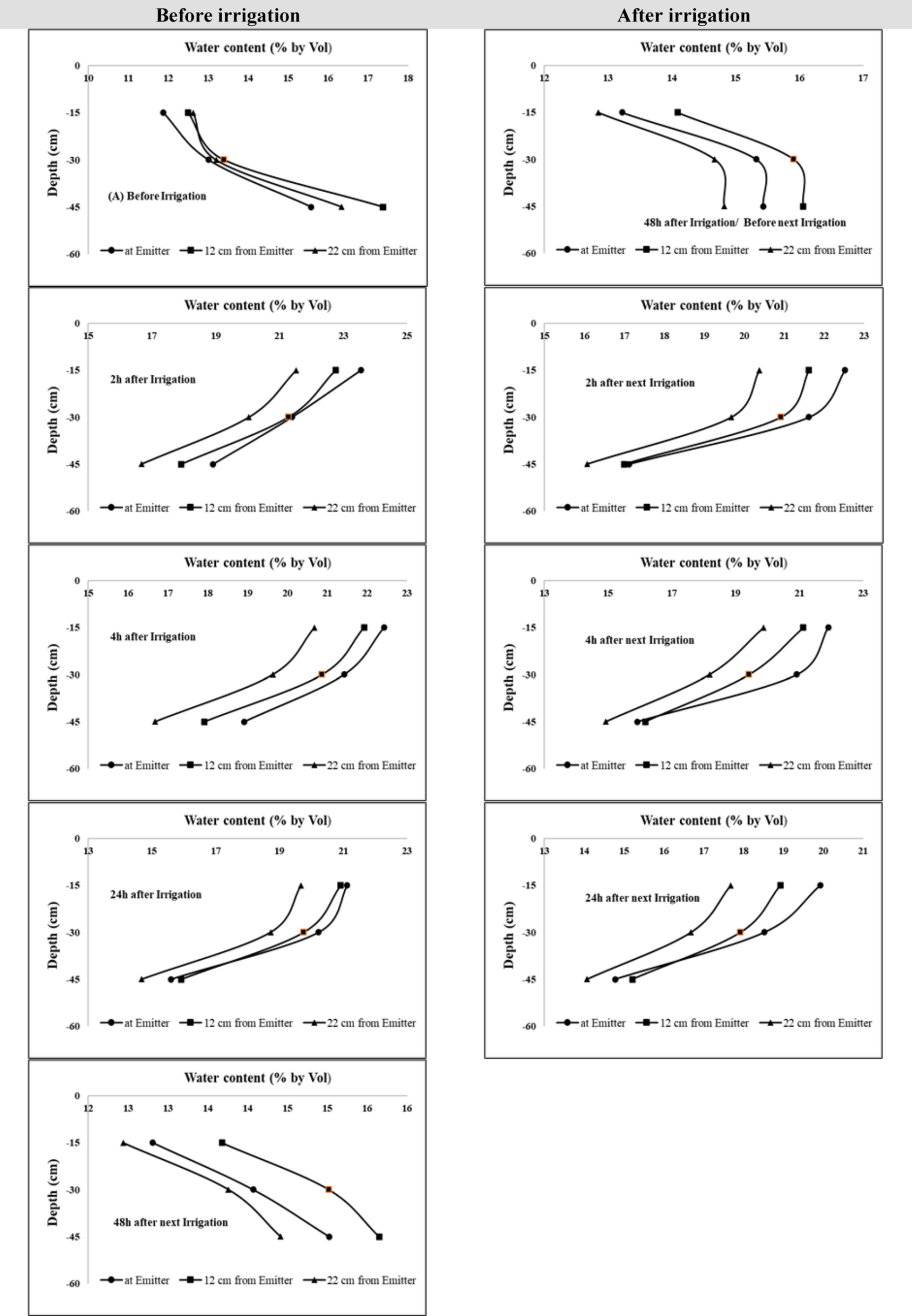
Average water distribution pattern for treatments *T*_*4*_, *T*_*5*_ and *T*_*6*_ two months after transplanting.

**Fig. 11:**
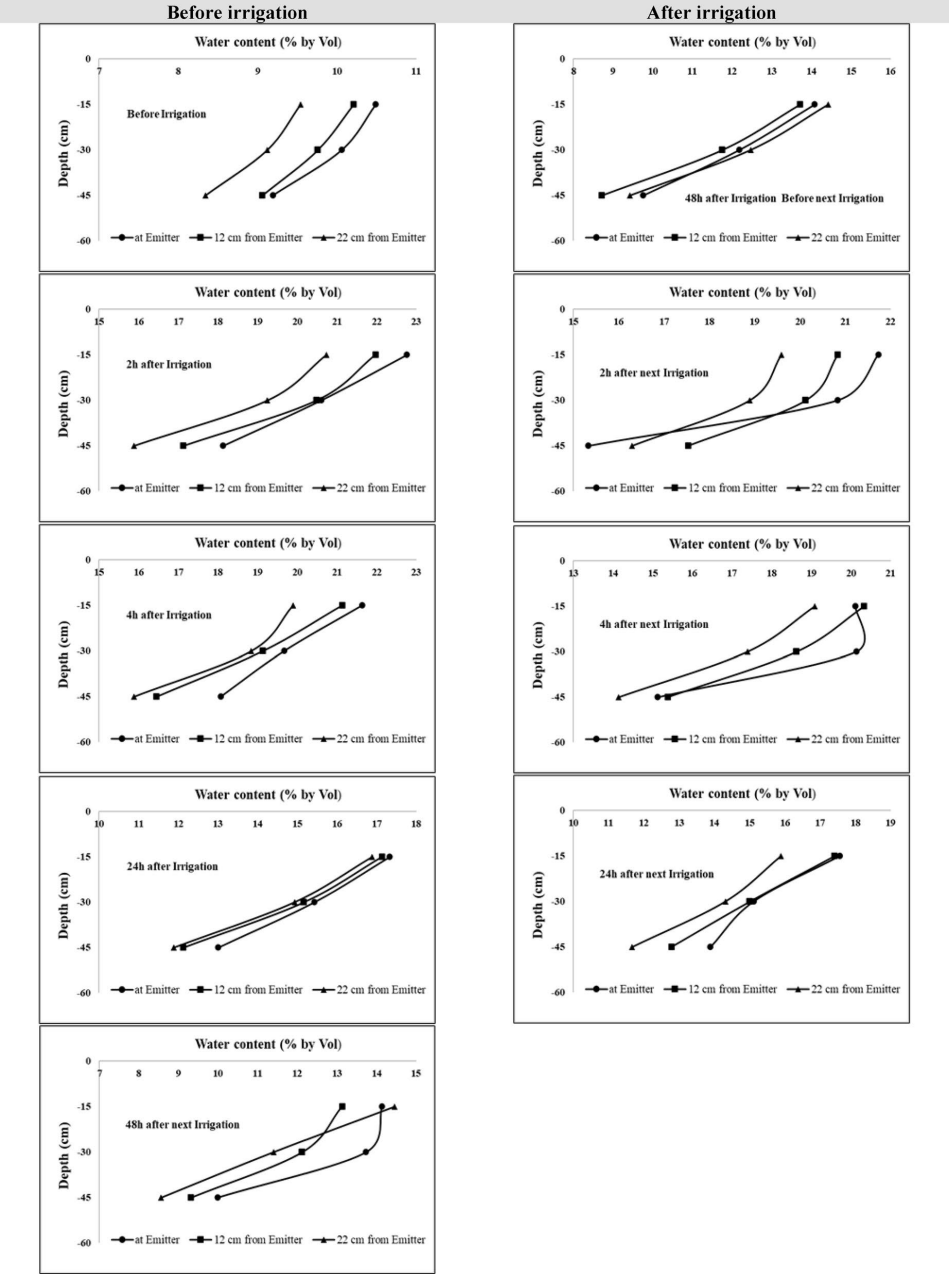
Average water distribution pattern for treatments *T*_*7*_, *T*_*8*_ and *T*_*9*_ two months after transplanting.

The results presented in this study include both field observations and simulation-based analyses. Field measurements were used for model calibration and validation, whereas simulation scenarios were conducted to evaluate the effects of varying emitter discharge rates and soil types on soil water distribution. The initial soil moisture content prior to transplanting chrysanthemum varied between 19.5% and 24% across soil layers. Immediately after transplanting, irrigation was applied to bring the soil close to field capacity to facilitate uniform crop establishment, and regular drip irrigation was initiated two days later. For analytical clarity, treatments were grouped by irrigation level (*T*_*1*_–*T*_*3*_, *T*_*4*_–*T*_*6*_, and *T*_*7*_–*T*_*9*_), and the mean soil moisture values for each group were used for comparison. The spatial and temporal dynamics of soil water distribution during the mid-season growth stage were evaluated at multiple intervals—before irrigation and at 2, 4, 24, and 48 h after irrigation, as well as corresponding intervals following the subsequent irrigation event (Figs. 9, 10 and 11).

Distinct wetting patterns were observed among emitter discharge (0.5 lph,1.0 lph, and 2.0 lph). Treatment *T*_*1*_ produced a relatively small wetted volume, whereas *T*_*3*_ generated extensive wetting but resulted in free drainage losses exceeding 12% (Table 4). In contrast, *T*_*2*_ provided the most uniform and effective wetting pattern, adequately covering the active root zone while minimizing percolation losses. Across all treatments, soil water content (*SWC*) was highest near the emitter and at the soil surface immediately after irrigation and declined with increasing depth and lateral distance, consistent with previously reported drip irrigation wetting behavior.

Treatments receiving 100% and 80% *ET*_*c*_ maintained higher *SWC* for longer durations compared with 60% *ET*_*c*_, where a limited water supply altered moisture distribution and reduced persistence. At 24 h after irrigation, *SWC* ranged from 17.36 to 23.68% beneath emitters and remained relatively uniform within the upper 0–30 cm root zone. By 48 h, moisture declined substantially, particularly under reduced irrigation. Since optimal chrysanthemum growth requires 17–23% *SWC*, favourable moisture conditions were sustained up to 48 h only under 100% *ET*_*c*_. Higher moisture depletion rates under deficit irrigation further emphasize the critical role of appropriate irrigation scheduling in maintaining adequate root-zone moisture and ensuring sustained crop water availability.

**Table 4:** Average rate of depletion of water (vol %) in 24 and 48 h in different treatments at one month after transplanting.

Depth (cm)	Average rate of depletion of water (volume, %)					
	24 h after irrigation			48 h after irrigation		
At emitter						
Treatments	***T***_***1***_, ***T***_***2***_, ***T***_***3***_	***T***_***4***_, ***T***_***5***_, ***T***_***6***_	***T***_***7***_, ***T***_***8***_, ***T***_***9***_	***T***_***1***_, ***T***_***2***_, ***T***_***3***_	***T***_***4***_, ***T***_***5***_, ***T***_***6***_	***T***_***7***_, ***T***_***8***_, ***T***_***9***_
0–15	2.43	2.43	5.43	15.78	10.33	8.68
15–30	2.17	1.17	5.17	19.98	6.07	8.42
30–45	4.12	3.32	5.12	21.05	3.49	8.37
12 cm from emitter						
0–15	1.84	1.84	4.84	16.65	8.67	8.25
15–30	0.84	1.52	5.32	18.47	5.37	8.73
30–45	1.00	2.00	5.00	19.65	1.86	8.41
22 cm from emitter						
0–15	1.74	1.84	3.84	14.08	8.67	6.30
15–30	1.79	1.32	4.32	17.48	5.37	6.78
30–45	2.23	2.00	4.00	18.48	1.86	6.46

As shown in Figs. 9, 10 and 11, *SWC* in the chrysanthemum root zone exceeded the capacity of field immediately after irrigation and remained close to field capacity for up to 24 h. By 48 h after irrigation, soil water content declined slightly below field capacity in the active root zone, indicating the need for irrigation at 48 h intervals. Thus, under sandy loam soil conditions inside the greenhouse, alternate-day irrigation is sufficient to maintain adequate root-zone moisture for chrysanthemum. Simulation results indicated that 48-hour irrigation intervals maintained SWC within the optimal range (0.22–0.30 cm^3^/cm^3^). Shorter intervals (< 24 h) increased drainage losses, whereas longer intervals (> 72 h) reduced *SWC* below 0.14 cm³/cm³, approaching the wilting point. Comparable patterns were observed across different growth stages, including the vegetative stage (1 month after transplanting), bud appearance stage (1.5 months), and harvesting stage (3 months). However, during the first month, soil moisture remained slightly higher within 4 h after irrigation. Similar trends were also evident at lateral distances of 12 cm and 22 cm from the emitter across all growth stages (vegetative, bud appearance, flowering, and harvesting).

### Calibration of inputs for water simulation in HYDRUS 2D and validation of the model

#### Optimization of soil hydraulic parameters through inverse modeling

Soil hydraulic parameters used for deriving *K*-*θ* relation by using Van Genuchaten model are represented in Table 5. They are *θ*_*r*_, *θ*_*s*_, *K*_*s*_, *α*, *n* and *I* (Tortuosity, dimensionless). Among these, values of *θ*_*r*_ and *θ*_*s*_, for distinct treatments utilized were those acquired from undisturbed samples obtained from the treatment plots. The values of *K*_s_, *α*, and *n* were obtained from the Rosetta Lite submodule, which used sand, silt, and clay percentages, along with bulk density (*BD*) and *SWC* at field capacity (0.33 bar) as inputs. These parameters were subsequently optimized through inverse modeling. The details of inverse modeling are given in material and methods section. It was observed that when using *K*_s_ values derived from Rosetta Lite, the model produced relatively high RMSE values. Therefore, measured *K*_s_ values obtained from the permeameter were used for optimization. Consequently, the Rosetta Lite-derived values of *α*, *n*, and *K*_s_ were treated as initial estimates, and appropriate minimum and maximum bounds were defined for these parameters. The time-variable atmospheric boundary conditions at the upper soil surface were specified, and the training dataset of *SWC* observations recorded during the simulation period was used to run the inverse model.

**Table 5:** Initial estimates and optimized values of soil hydraulic parameters obtained from inverse modeling.

Soil depth (cm)	θ_*r*_ (m^3^/m^3^)	θ_s_ (m^3^/m^3^)	α (1/cm)	*N*	K_s_ (cm/h)	I
Initial estimated/ measured values						
0–15	0.065	0.413	0.075	1.89	3.462	0.5
15–30	0.052	0.396	0.028	1.46	1.793	0.5
30–45	0.049	0.402	0.030	1.42	1.985	0.5
Optimized values through inverse modeling						
0–15	**-**	**-**	0.053	1.73	3.937	**-**
15–30	**-**	**-**	0.031	1.32	1.460	**-**
30–45	**-**	**-**	0.042	1.56	2.579	**-**

#### Time variable boundary conditions

**Fig. 12:**
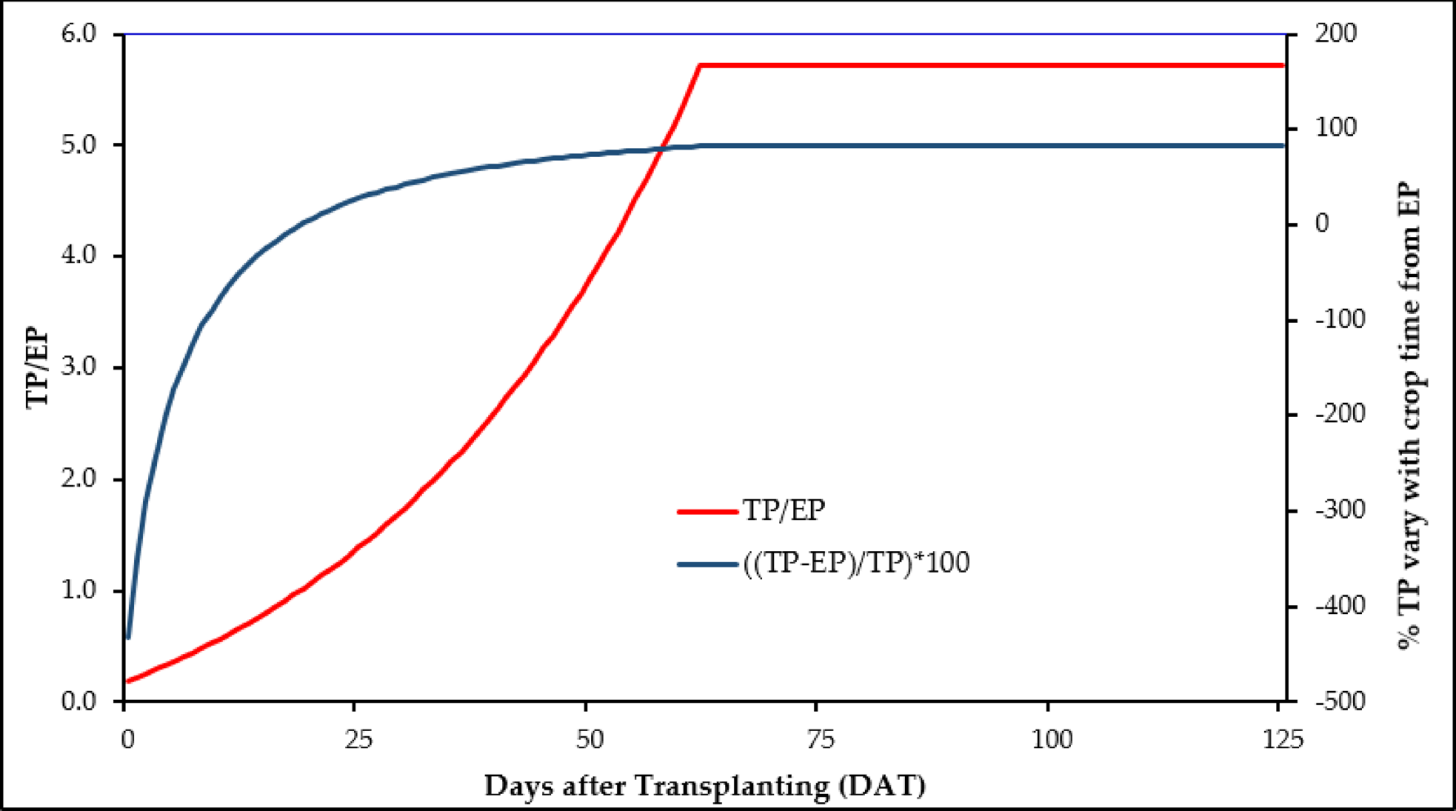
Variation of EP/TP during 126 days of planting.

The partitioning of *ET*_*c*_ into *EP* and *TP* was performed to distinguish unproductive (*EP*) from productive (*TP*) fluxes. These components were subsequently used as time-dependent input parameters in HYDRUS-2D simulations. As shown in Fig. 12, the ratio of *TP/EP* increased progressively until 61 days after transplanting (*DAT*), after which it stabilized at approximately 5.72 and remained constant until harvest. This indicates that TP increased steadily as the crop progressed toward full maturity, reaching maximum at mid-season stage, then leveled off. Notably, *TP* surpassed *EP* after 19–20 *DAT*, while prior to this period, *EP* exceeded *TP*. Initially, TP was about 435% lower than *EP*, but by the end of the cropping cycle, it exceeded *EP* by nearly 85%.

#### Root growth parameters as input to root water uptake simulation

Root growth parameters required as inputs for simulating root water uptake (RWU) utilizing the Feddes et al.^75^ method included maximum rooting depth, depth of maximum root intensity, maximum horizontal rooting radius (i.e., the lateral extent of roots on either side of the plant base), and the radius of maximum horizontal root intensity (average root radius in cm). For all treatments, the total rooting depth was assumed to be 45 cm. In treatment *T*_*2*_, the maximum root length density (*RLD*) was observed at a depth of 12.6 cm. At the flowering stage, the maximum horizontal root spread and maximum root radius were 7.5 cm and 0.047 cm, respectively.

**Fig. 13:**
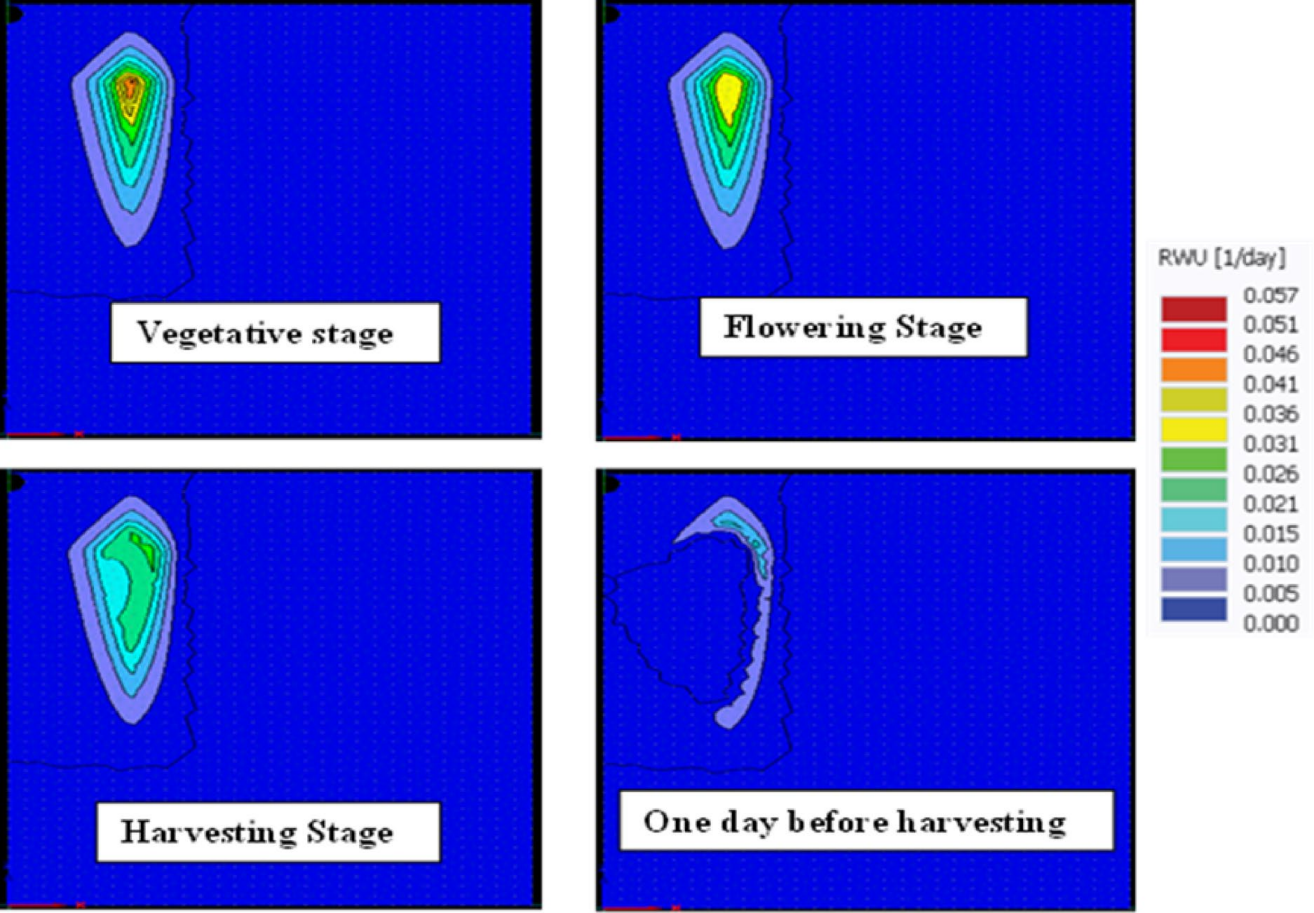
Pictorial presentation of RWU under sandy loam soil (Emitter discharge – 1 lph) on different days during simulation period.

A pictorial representation of simulated RWU across the soil profile under sandy loam conditions at different growth stages is shown in Fig. 13. The figure illustrates water uptake patterns at the vegetative, flowering, and harvesting stages, as well as one day prior to harvest. Results revealed that *RWU* was highest during the vegetative stage, followed by the flowering stage, and lowest during the harvesting stage, reflecting a progressive decline in crop water demand as maturity advanced.

#### Calibration and validation of the model

**Fig. 14:**
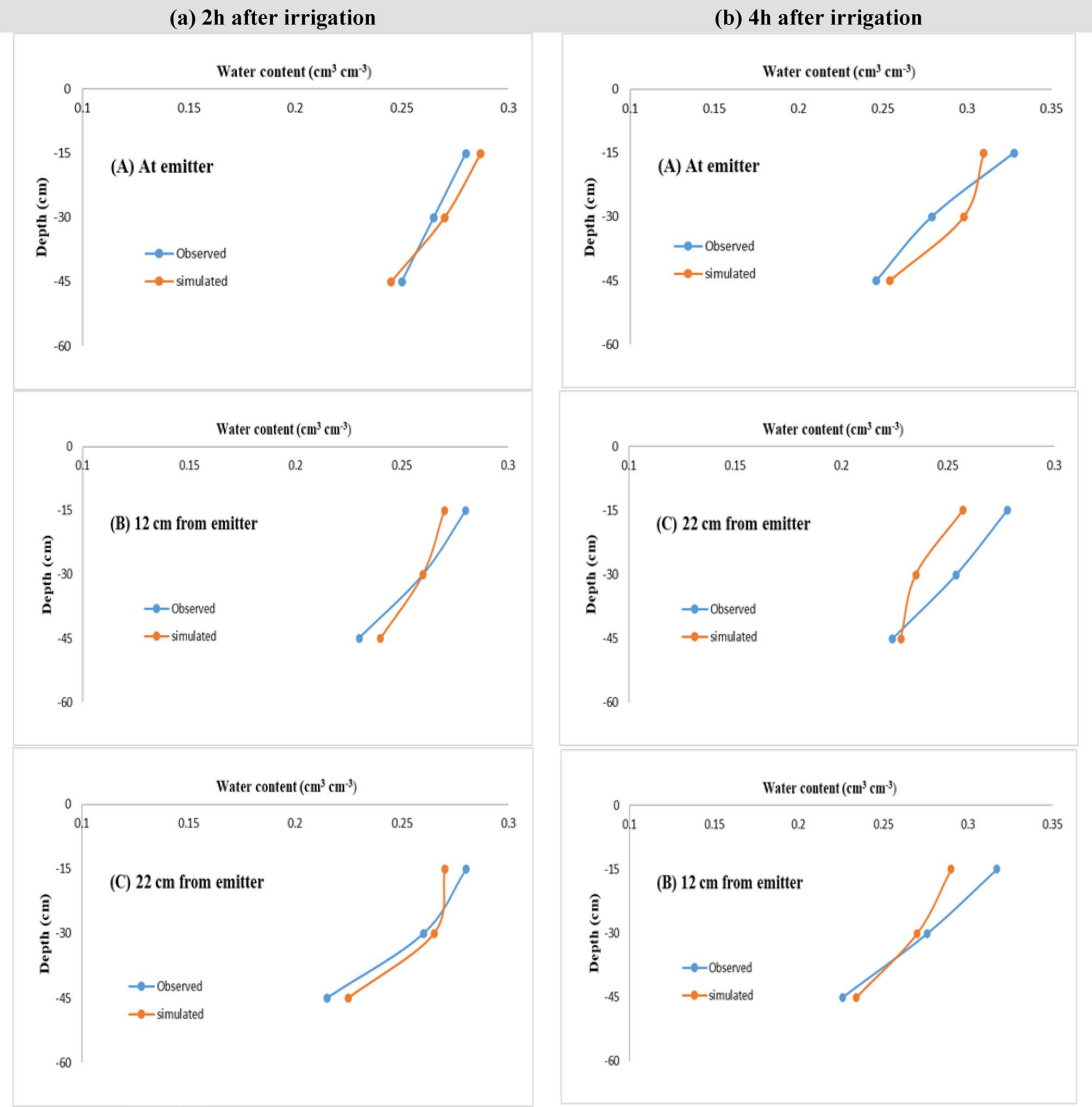
Simulated and observed water content (**a**) at the end of the first month after transplanting and 2 h after irrigation and (**b**) at the end of the first month after transplanting and 4 h after irrigation. (**a**) 2 h after irrigation, (**b**) 4 h after irrigation.

The HYDRUS-2D model was calibrated and validated utilizing observed SWC from treatment *T*_*2*_ to predict water distribution in the root zone of chrysanthemum. Model inputs were obtained from field experiments and published sources, with particular emphasis on hydraulic conductivity and dispersity of sandy loam soil. Calibration was performed using measured hydraulic conductivity values, and results demonstrated satisfactory agreement between simulated and observed water contents. Graphical outputs from HYDRUS-2D were processed to generate SWC profiles as a function of depth, and field observations were collected at the end of the first month after transplanting, at multiple intervals (2, 4, 12, 24, 48, 50, 52, and 72 h after irrigation). Comparisons showed that simulated and observed values followed similar trends with minimal deviation (Fig. 14(a-b)).

**Fig. 15:**
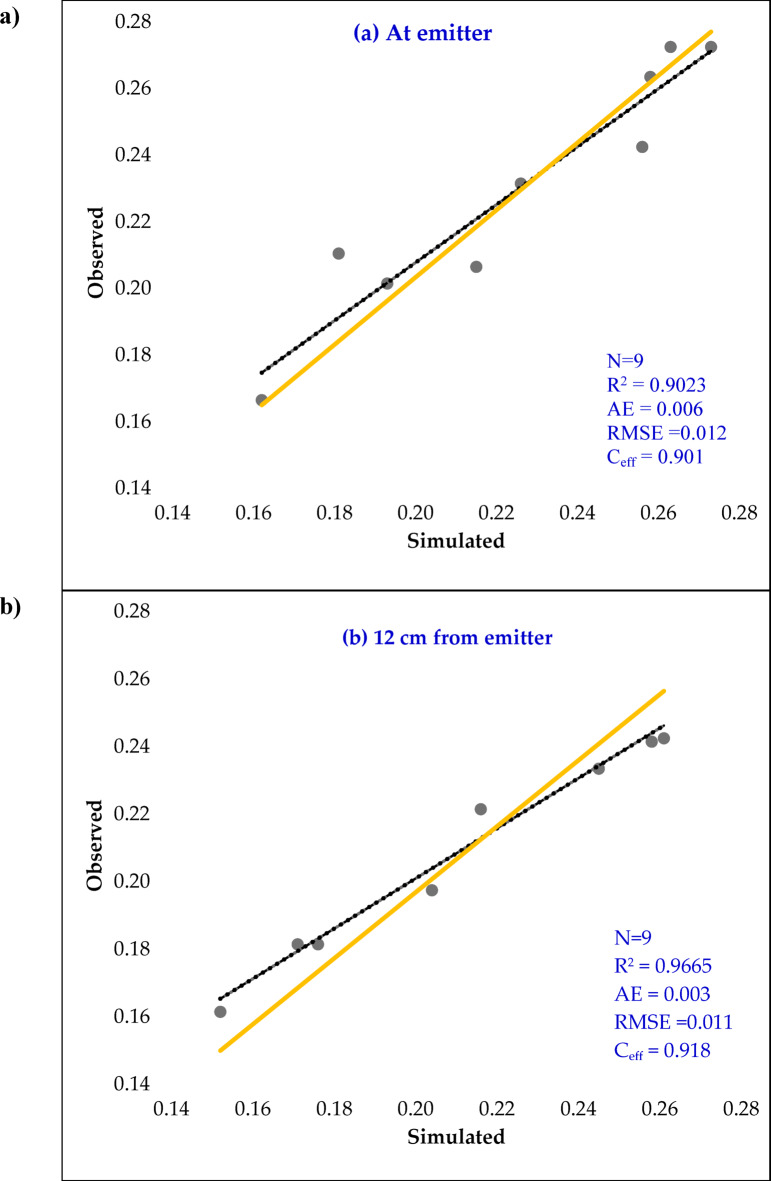

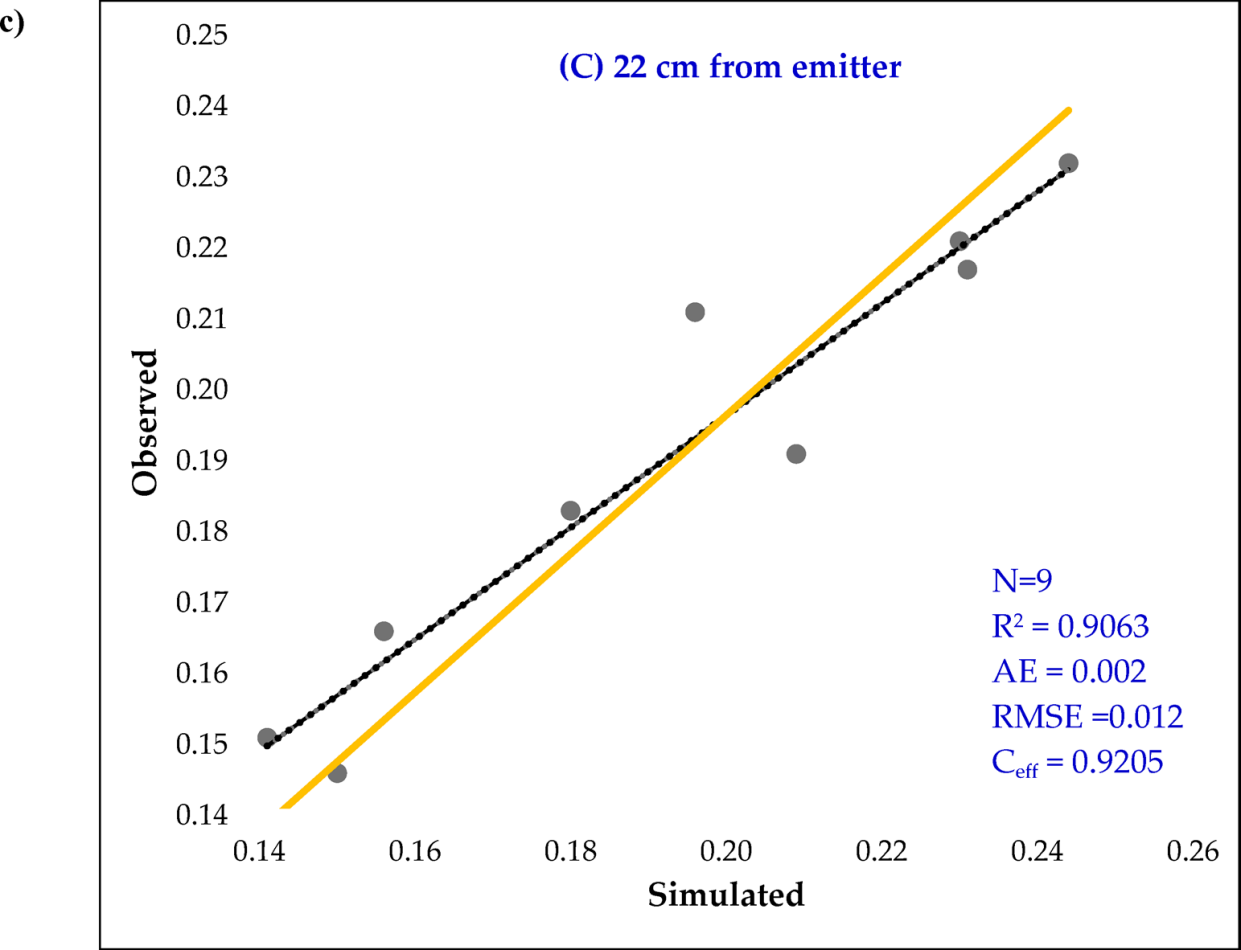
Comparison of observed and predicted *SWC* (cm^3^/cm^3^) of the model for Treatment *T*_*2*_ in the first two irrigation cycles during the flowering stage of the crop at 12 cm from the emitter (below the plant); (**a**) At emitter, (**b**) 12 cm from emitter, (**c**) 22 cm from emitter.

Model validation was carried out at the flowering stage using nine independent datasets collected at the emitter, 12 cm, and 22 cm lateral distances (Fig. 15). The statistical evaluation confirmed high predictive performance. The *R*^*2*^ between observed and simulated water contents ranged from 0.90 to 0.96, indicating strong correlation. The *RMSE* values were low, ranging from 0.011 to 0.012, while the average error (*AE*) ranged from 0.006 to 0.02. The *C*_*eff*_ was also high, ranging between 0.90 and 0.92. These results confirm that HYDRUS-2D reliably captured soil water distribution under drip irrigation, demonstrating its suitability as a robust tool for simulating water dynamics in chrysanthemum cultivation.

#### Simulated water distribution

After successful calibration and validation, HYDRUS-2D was employed to simulate soil water distribution across a range of soil textures, namely sandy loam, sandy clay loam, loam, silty clay loam, and silt, under four emitter discharge rates (1.0, 1.5, 2.0, and 2.5 lph). A total of sixty simulation scenarios, combining different soil types, discharge rates, and fertigation strategies, were evaluated to assess the spatiotemporal distribution of water within the root zone.

#### Effect of soil type on water distribution

**Fig. 16:**
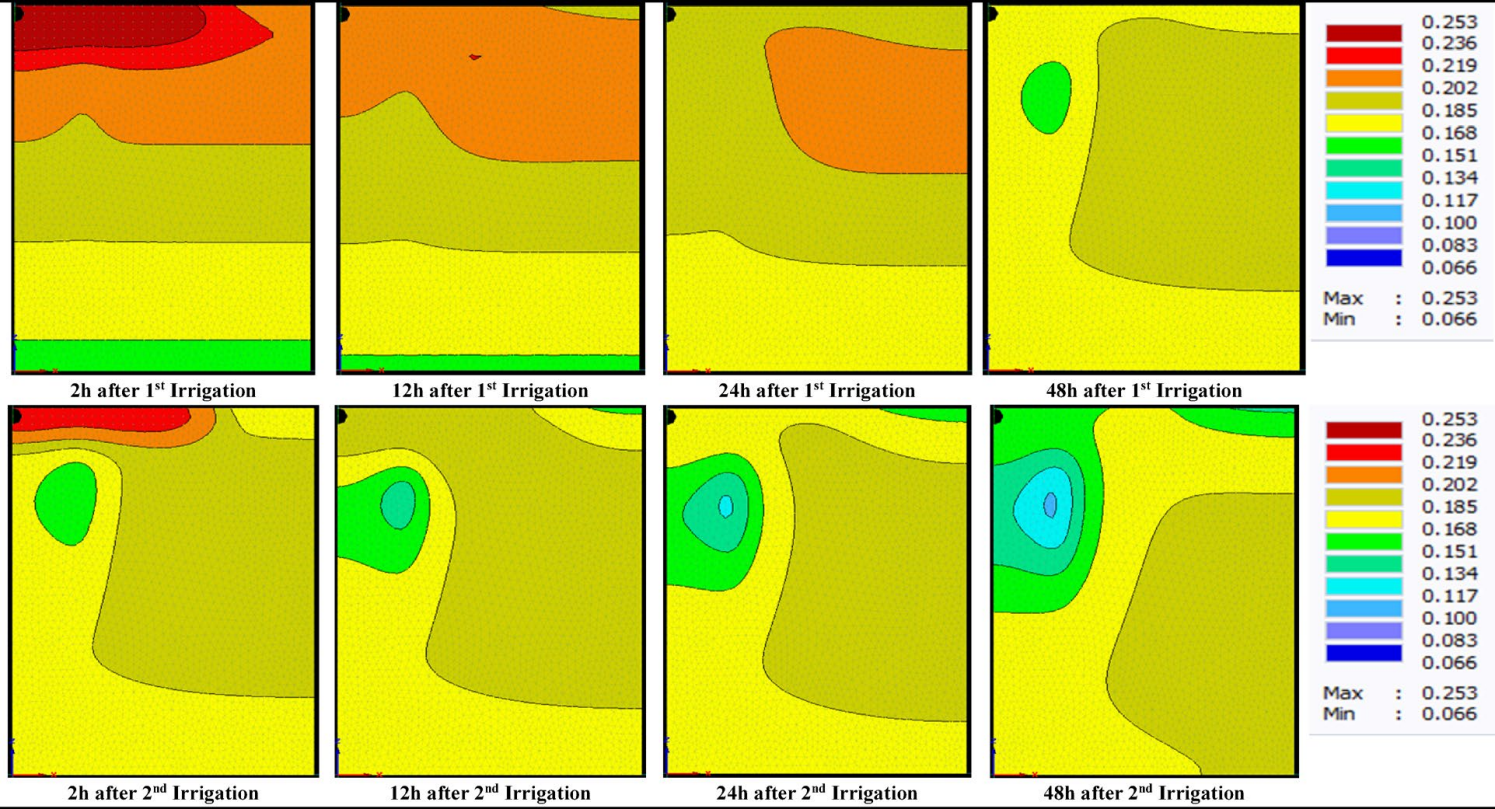
Simulated soil and water distribution in sandy loam soil (Emitter discharge – 1 lph).

**Fig. 17:**
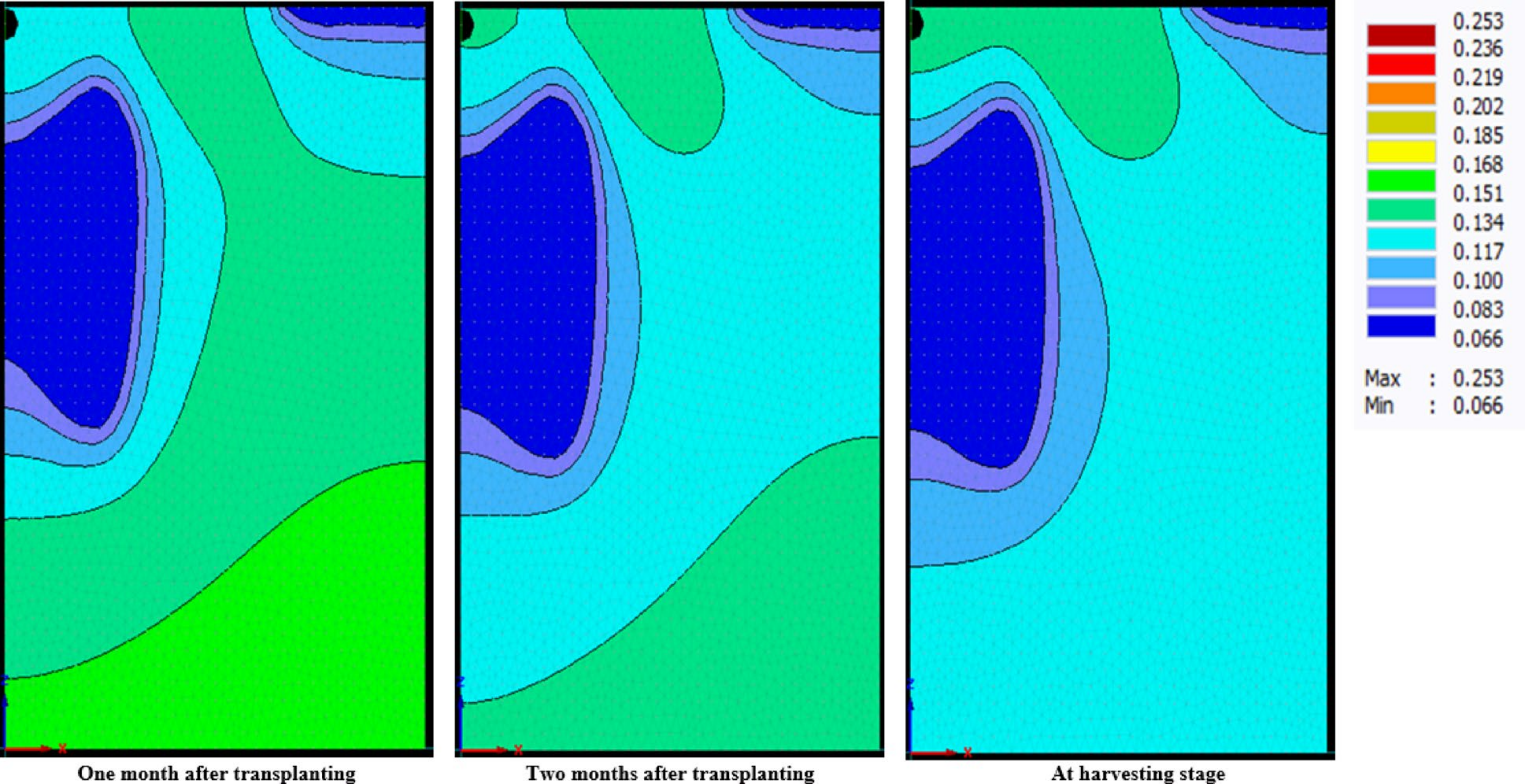
Simulated soil and water distribution in sandy loam soil (Emitter discharge – 1 lph).

The influence of soil type on water distribution was analyzed by considering spatial moisture variation at different times after irrigation, free drainage losses below the root zone, and the advancement of the wetting front. Spatial distribution of SWC at the onset of the first and second irrigations (i.e., after two irrigation cycles) with an emitter discharge of 1.0 lph at the end of the second month for sandy loam soil is illustrated in Fig. 16 through calibrated and validated color-spectrum outputs. Initial soil moisture content was maintained uniformly across layers in all soils. Pictorial simulations of sandy loam soil at one month, two months, and at harvest are presented in Fig. 17. Results indicate that in sandy loam, higher permeability led to lower water content in upper layers compared with less permeable soils, while deeper layers retained more water. Radial uniformity was observed in sandy loam, whereas other soils exhibited declining moisture with distance from the emitter.

The comparative analysis of soil water distribution demonstrated distinct differences across soil types. In sandy loam, which is more permeable, higher water content was measured in the middle soil layer compared with the less permeable sandy clay loam. Across all soils, however, water content remained relatively uniform and sufficient within the root zone up to 48 h after irrigation, thereby ensuring an adequate moisture supply for crop growth. Within the emitter vicinity (10 cm radius), moisture content followed the order: silt ≈ silty clay loam > loam > sandy clay loam > sandy loam. Simulations of water distribution were conducted under identical conditions with an emitter discharge of 1.0 lph, and comparisons were made at multiple intervals (2, 12, 24, and 48 h after the first and second irrigations, as well as at one month, two months, and harvest stage).

The results indicated that SWC in the top layer decreased more rapidly over time compared with deeper layers, with sandy loam showing the fastest depletion due to its higher permeability. At 12 h after irrigation, water content near the emitter was greatest in silty soils (0.207 in silt and 0.264 in silty clay loam), followed by loam, sandy loam, and sandy clay loam. In the middle layer, water content was generally similar across soil types, except for sandy loam, which retained less water.

Spatial distributions illustrated through color spectra (Figs. 16 and 17) revealed that the applied flux extended to the deepest layer (30–45 cm) across all soils, as indicated by higher post-irrigation water content compared with initial values. This suggests downward percolation beyond the root zone, reducing plant-available water. In sandy loam and sandy clay loam, upper-layer water content remained 0.24 and 0.28, respectively, even after 24 h, confirming that water infiltration was faster in sandy loam, resulting in lower surface moisture retention relative to less permeable soils.

### Effect of emitter discharge on water distribution

The volume of water percolating below the root zone (simulated depth of 45 cm) was estimated from the cumulative free drainage boundary flux generated by HYDRUS-2D simulations. For each soil type, cumulative free drainage was expressed as a percentage of applied irrigation water (Table 6). Results indicated that free drainage losses were negligible in silty clay loam soils at emitter discharges of 1.0 and 1.5 lph, followed by silt, loam, sandy clay loam, and sandy loam. The effect of emitter discharge on free drainage was minimal in silty clay loam and silt soils, reflecting their lower permeability. By contrast, free drainage increased with higher discharge rates in more permeable soils. Specifically, when emitter discharge increased from 1.0 to 2.5 lph, free drainage rose from 426.01 to 477.42 cm^3^ in sandy clay loam, 1145.82 to 1263.34 cm^3^ in sandy loam, 337.87 to 387.82 cm^3^ in loam, and 88.14 to 96.87 cm^3^ in silt soils. The increment was most pronounced in sandy loam, confirming its higher susceptibility to deep percolation losses. Overall, the findings demonstrate that soil hydraulic properties exert a stronger influence on free drainage than emitter discharge does, particularly in less-permeable soils such as silt and silty clay loam. These insights are critical for designing and operating drip irrigation systems to minimize non-productive water losses below the root zone.

**Table 6:** Simulated free drainage (cm^3^) (% of applied irrigation water) below 45 cm depth under different soil types and emitter discharge rates.

Soil type	Emitter discharge (lph)			
	1.0	1.5	2.0	2.5
Sandy clay loam	426.01 (0.29)	434.82 (0.295)	440.70 (0.30)	477.42 (0.325)
Sandy loam	1145.82 (0.78)	1189.89 (0.81)	1219.27 (0.83)	1263.34 (0.86)
Loam	337.87 (0.23)	345.21 (0.235)	352.56 (0.24)	387.82 (0.264)
Silt	88.14 (0.06)	90.17 (0.065)	96.28 (0.067)	96.87 (0.068)
Silty clay loam	Neg.	Neg.	44.07 (0.03)	54.35 (0.037)

Values are expressed as volume (cm^3^), with percentages in parentheses indicating the proportion of applied irrigation water.

## Discussion

A finite element model, HYDRUS-2D, was employed to simulate soil water movement within the root zone of chrysanthemum under drip irrigation. The simulations were conducted in an axi-symmetrical polar coordinate system with a radial extent of 30 cm (representing half of the lateral-to-lateral spacing) and a depth of 45 cm, corresponding to the effective rooting depth of chrysanthemum. Multiple scenarios were simulated by combining different soil types and emitter discharge rates to capture variability in water distribution under greenhouse conditions. The strong agreement between simulated and observed SWC (*R*^*2*^ = 0.90–0.96, *RMSE* = 0.011–0.012 cm^3^ cm^-3^, *C*_*eff*_ = 0.90–0.92) confirms the reliability of HYDRUS-2D for predicting soil water dynamics under sandy loam soils in greenhouse conditions. These outcomes are consistent with previous studies that have successfully applied HYDRUS-based models to simulate water movement under drip irrigation systems. Irrigation scheduling and emitter discharge were found to significantly influence soil water distribution and availability. The soil moisture remained near field capacity for up to 24 h after irrigation, then declined gradually thereafter. In 48 h, soil moisture approached the lower threshold of the optimal range for chrysanthemum growth, indicating that an irrigation interval of 48 h is appropriate for maintaining adequate root-zone moisture under sandy loam conditions. Shorter irrigation intervals increased the likelihood of deep percolation losses, whereas longer intervals resulted in moisture depletion below desirable levels. Soil hydraulic properties played a critical role in determining water distribution patterns. Finer-textured soils such as silt and silty clay loam exhibited higher water retention near the emitter, resulting in more uniform moisture distribution within the upper soil layers. In contrast, coarser soils such as sandy loam showed faster infiltration and deeper percolation, leading to comparatively lower moisture retention in the surface layers. This behavior is consistent with the fundamental principles of soil physics, where pore size distribution governs water retention and movement. The observed differences across soil types emphasize the need for soil-specific irrigation design to improve water-use efficiency. The effect of emitter discharge on water distribution further reinforced the importance of optimizing irrigation design. Lower discharge rates resulted in limited wetted volume, potentially restricting water availability in deeper root zones. Conversely, higher discharge rates increased the wetted area but also led to greater deep percolation losses, particularly in more permeable soils. The intermediate discharge rate (1.0 lph) provided the most balanced performance by ensuring adequate wetting of the active root zone while minimizing drainage losses.

For modeling root water uptake (*RWU*), the current study applied the Feddes et al.^75^ uncompensated root water uptake model, which is a macroscopic approach. This method is widely used in vadose zone modeling studies as it simplifies the complex root–soil interactions by distributing potential transpiration across the root zone proportionally to root density^76^. Soil water stress functions are then applied to locally reduce uptake depending on the soil matric potential or salinity conditions^77^. The modeling framework adopted in this study relies on certain simplifying assumptions. Root distribution was considered static and based on measurements taken at the flowering stage, representing peak root development. While this assumption is commonly adopted in HYDRUS simulations, it does not capture the dynamic nature of root growth over the entire crop cycle. Similarly, root water uptake was modeled using the macroscopic Feddes approach, which simplifies complex soil–plant interactions by distributing water uptake according to root density and soil water pressure head. Although these assumptions limit the representation of detailed root processes, provides a practical and computationally efficient approach for field-scale simulation under controlled conditions. Despite these simplifications, the model effectively captured the key dynamics of soil water movement and root-zone moisture availability. The integration of field observations with numerical simulation enabled a comprehensive understanding of irrigation performance under varying conditions.

Previous applications of the HYDRUS model have extensively demonstrated its capability to simulate soil water dynamics across a range of agricultural systems, including subsurface drip irrigation and controlled environments. Provenzano^49^ applied HYDRUS-2D to evaluate wetted soil volume under subsurface drip irrigation, primarily focusing on axisymmetric infiltration around a single emitter and its implications for irrigation design. Similarly, Vital et al.^55^ employed HYDRUS (2D/3D) to investigate soil moisture dynamics under contrasting tillage practices, emphasizing inverse modeling and continuous monitoring of soil water content and matric potential. While these studies confirm the robustness of HYDRUS for simulating water flow processes, they are largely oriented toward model validation, infiltration characterization, or system-specific applications under controlled or field-scale conditions. In contrast to previous studies, this research systematically examines soil wetting behavior under different emitter discharge rates and irrigation intervals through the integration of field measurements and HYDRUS-2D simulations providing practical guidance for irrigation scheduling in protected chrysanthemum cultivation across different soil textures.This approach provides deeper insight into the spatiotemporal evolution of soil moisture under point-source drip irrigation, thereby addressing limitations in previous studies that did not explicitly link HYDRUS simulations with design-oriented evaluation of wetting geometry under variable operational conditions. Recent studies have demonstrated that HYDRUS-based simulations can effectively link water and nutrient transport processes with management strategies and environmental risk assessment. Liang et al.^78^ showed that optimized fertilization practices can reduce nutrient losses while improving crop productivity, highlighting the potential of process-based modeling to support sustainable agricultural decision-making. Studies on soil water movement under irrigation not only improve understanding of wetting dynamics but also provide a basis for extending analyses toward solute transport and fertigation processes. Ashrafi et al.^79^ demonstrated the application of analytical modeling approaches to simulate solute transport in furrow fertigation systems, highlighting the broader relevance of water flow studies for nutrient transport and environmental assessments.

This study provides a practical framework for optimizing drip irrigation design and management in chrysanthemum cultivation under protected conditions. Future research may focus on incorporating dynamic root growth, three-dimensional modeling, and multi-season validation to further enhance model accuracy and applicability. Such advancements would support the development of more precise and adaptive irrigation strategies for sustainable horticultural production systems.

## Conclusions

This study demonstrates the effectiveness of the HYDRUS-2D model as a robust tool for simulating soil water dynamics under drip fertigation in chrysanthemum cultivated under protected conditions. By integrating field experimentation with numerical simulation, the study provides a comprehensive assessment of root-zone moisture behavior across varying soil textures, irrigation regimes, and emitter discharge rates. The results clearly indicate that irrigation scheduling and soil hydraulic properties play a decisive role in determining water availability and distribution within the root zone. An emitter discharge of 1.0 lph combined with a 48-hour irrigation interval was found to be optimal for maintaining soil moisture within the desirable range while minimizing deep percolation losses under sandy loam conditions. Finer-textured soils exhibited higher moisture retention near the emitter, whereas coarser soils promoted deeper water movement, underscoring the need for soil-specific irrigation strategies. Importantly, the study moves beyond conventional model validation by translating HYDRUS-2D simulation outputs into practical irrigation design recommendations for chrysanthemum. This application-oriented approach enhances the utility of simulation modeling as a decision-support tool for greenhouse irrigation management. Although the modeling framework incorporates simplified assumptions, including static root distribution and a macroscopic root water uptake formulation, it effectively captures the dominant processes governing soil water movement under drip irrigation. These assumptions provide a pragmatic balance between model complexity and applicability under field conditions. Overall, the findings contribute to improving water-use efficiency and irrigation planning in protected floriculture systems. The approach presented in this study can be extended to other crops and agro-climatic conditions, offering a scalable framework for sustainable water management in intensive horticultural production systems.

## Data Availability

The data that support the findings of this study are available on request from the corresponding author. The data are not publicly available due to privacy or ethical restrictions.

## References

[1] Debnath, S. & Peera, P. G. S. K. Irrigation and fertigation in protected cultivation. In *Protected Cultivation and Smart Agriculture* (eds Maitra, S., Gaikwad, J. & Shankar, T. D.) 40–54 10.30954/NDP-PCSA.2020.5. (2020).

[2] Wang, H. et al. Optimal drip fertigation management improves yield, quality, water and nitrogen use efficiency of greenhouse cucumber. *Sci. Hortic.* **243**, 357–366 (2019).

[3] Vishwakarma, D. K. et al. Green house technology for controlled environment-advances in green house automation and control. In *Greenhouse Technology for Sustainable Agriculture* (eds Bhat, S. A., Amin, T., Bashir, O. & Khan, S. A.) 111–139 10.1201/9781003455967-5. (Apple Academic, 2025).

[4] Hasan, M. & Sirohi, N. P. S. Automation of irrigation system in protected cultivation. *Agric. Eng. Today*. **27**, 63–70 (2003).

[5] Marcelis, L. F. M. et al. The adaptive greenhouse-an integrated systems approach to developing protected cultivation systems. In *III International Symposium on Models for Plant Growth, Environmental Control and Farm Management in Protected Cultivation*. 718 399–406 (2006).

[6] Prasanna, R. et al. Chrysanthemum growth gains from beneficial microbial interactions and fertility improvements in soil under protected cultivation. *Hortic. Plant. J.* **2**, 229–239 (2016).

[7] Bharti, A. et al. Cyanobacterial amendment boosts plant growth and flower quality in Chrysanthemum through improved nutrient availability. *Appl. Soil. Ecol.* **162**, 103899 (2021).

[8] Corradini, C. Soil moisture in the development of hydrological processes and its determination at different spatial scales. *J. Hydrol. (Amst)*. **516**, 1–5 (2014).

[9] Yin, H., Bista, P., Ghimire, R., Yang, H., & Carroll, K. C. (2026). Unraveling soil moisture dynamics with dual-scale interpretable machine learning: Cover cropping and irrigation insights in semi-arid agriculture. Vadose Zone Journal, 25(1), e70077. doi: 10.1002/vzj2.70077

[10] Yang, J. et al. Global Soil Water Stable Isotope Dataset. Scientific Data. (2026). 10.1038/s41597-026-07262-8 10.1038/s41597-026-07262-8 PMC13276124 42000782

[11] Chen, Y. et al. Mulching practices altered soil bacterial community structure and improved orchard productivity and apple quality after five growing seasons. *Sci. Hortic.* **172**, 248–257 (2014).

[12] Chen, M., Willgoose, G. R. & Saco, P. M. Spatial prediction of temporal soil moisture dynamics using HYDRUS-1D. *Hydrol. Process.* **28**, 171–185 (2014).

[13] Elmaloglou, S. T. & Malamos, N. Estimation of width and depth of the wetted soil volume under a surface emitter, considering root water-uptake and evaporation. *Water Resour. Manag*. **21**, 1325–1340 (2007).

[14] Kumar, R. & Kumar, M. Effect of drip irrigated mulch on soil properties and water use efficiency-A review. *J. Soil. Water Conserv.* **19**, 300 (2020).

[15] Vishwakarma, D. K. et al. Evaluation and development of empirical models for wetted soil fronts under drip irrigation in high-density apple crop from a point source. *Irrig. Sci.*10.1007/s00271-022-00826-7 (2022).

[16] Kumar, M., Kumar, R., Rajput, T. B. S. & Patel, N. Efficient design of drip irrigation system using water and fertilizer application uniformity at different operating pressures in a semi-arid region of India. *Irrig. Sci.* **66**, 316–326 (2017).

[17] Sun, L. et al. Simulation of soil water movement and root uptake under mulched drip irrigation of greenhouse tomatoes. *Water (Basel)*. **15**, 1282 (2023).

[18] Slama, F., Zemni, N., Bouksila, F., De Mascellis, R. & Bouhlila, R. Modelling the impact on root water uptake and solute return flow of different drip irrigation regimes with brackish water. *Water (Basel)*. **11**, 425 (2019).

[19] Phull, A. M. & Babar, M. M. Summulation of soil wetting pattern of subsurface. In *Sixteenth International Water Technology Conference*. 16 1–11 (IWTC, Istanbul, Turkey, 2012).

[20] Al-Ogaidi, A. A. M., Aimrun, W., Rowshon, M. K. & Abdullah, A. F. WPEDIS – wetting pattern estimator under drip irrigation systems. In *International Conference on Agricultural and Food Engineering* 198–203 (Cafei, 2016).

[21] Malek, K. & Peters, R. T. Wetting pattern models for drip irrigation: New empirical model. *J. Irrig. Drain. Eng.* **137**, 530–536 (2011).

[22] Ainechee, G., Boroomand-, S. & Behzad, M. Simulation of soil Wetting pattern under point source trickle irrigation. *J. Appl. Sci.* **9**, 1170–1174 (2009).

[23] Liu, Z., Li, P., Hu, Y. & Wang, J. Modeling the wetting patterns in cultivation substrates under drip irrigation. *J. Coast Res.* **73**, 173–176 (2015).

[24] Zhang, Y. Y., Zhao, X. N. & Wu, P. Te. Soil wetting patterns and water distribution as affected by irrigation for uncropped ridges and furrows. *Pedosphere* **25**, 468–477 10.1016/S1002-0160(15)30014-X (2015).

[25] Vishwakarma, D. K. et al. Modeling of soil moisture movement and wetting behavior under point-source trickle irrigation. *Sci. Rep.* **13**, 14981 (2023). 37696862 10.1038/s41598-023-41435-4 PMC10495428

[26] Azad, N., Behmanesh, J., Rezaverdinejad, V., Abbasi, F. & Navabian, M. Developing an optimization model in drip fertigation management to consider environmental issues and supply plant requirements. *Agric. Water Manag*. **208**, 344–356 (2018).

[27] Phogat, V., Mahadevan, M., Skewes, M. & Cox, J. W. Modelling soil water and salt dynamics under pulsed and continuous surface drip irrigation of almond and implications of system design. *Irrig. Sci.* **30**, 315–333 (2012).

[28] Han, M., Zhao, C., Feng, G., Yan, Y. & Sheng, Y. Evaluating the effects of mulch and irrigation amount on soil water distribution and root zone water balance using HYDRUS-2D. *Water (Switzerland)*. **7**, 2622–2640 (2015).

[29] Kandelous, M. M. & Šimůnek, J. Numerical simulations of water movement in a subsurface drip irrigation system under field and laboratory conditions using HYDRUS-2D. *Agric. Water Manag*. **97**, 1070–1076 (2010).

[30] Kandelous, M. M. & Šimůnek, J. Comparison of numerical, analytical, and empirical models to estimate wetting patterns for surface and subsurface drip irrigation. *Irrig. Sci.* **28**, 435–444 (2010).

[31] Skaggs, T. H., Trout, T. J., Šimůnek, J. & Shouse, P. J. Comparison of HYDRUS-2D simulations of drip irrigation with experimental observations. *J. Irrig. Drain. Eng.* **130**, 304–310 (2004).

[32] Sun, X., Tong, J., Liu, C., Ma, Y. & Using HYDRUS-2D model to simulate the water flow and nitrogen transport in a paddy field with traditional flooded irrigation. *Environ. Sci. Pollut. Res.* **29**, 32894–32912 (2022). 10.1007/s11356-021-18457-4 35020147

[33] Singh, M. C., Jain, A. K. & Garg, S. Simulation of soil moisture movement under rice field using Hydrus-2D. *Crop Res. (Hisar)*. **45**, 45–53 (2013).

[34] Wongkaew, A., Saito, H., Fujimaki, H. & Šimůnek, J. Numerical analysis of soil water dynamics in a soil column with an artificial capillary barrier growing leaf vegetables. *Soil. Use Manag*. **34**, 206–215 (2018).

[35] Siyal, A. A. & Skaggs, T. H. Measured and simulated soil wetting patterns under porous clay pipe sub-surface irrigation. *Agric. Water Manag*. **96**, 893–904 (2009).

[36] Skaggs, T. H., Trout, T. J. & Rothfuss, Y. Drip irrigation water distribution patterns: Effects of emitter rate, pulsing, and antecedent water. *Soil Sci. Soc. Am. J.* **74**, 1886–1896 (2010).

[37] Lazarovitch, N., Warrick, A. W., Furman, A. & Šimůnek, J. Subsurface water distribution from drip irrigation described by moment analyses. *Vadose Zone J.* **6**, 116–123 (2007).

[38] Hinnell, A. C., Lazarovitch, N., Furman, A., Poulton, M. & Warrick, A. W. Neuro-Drip: estimation of subsurface wetting patterns for drip irrigation using neural networks. *Irrig. Sci.* **28**, 535–544 (2010).

[39] Lazarovitch, N., Poulton, M., Furman, A. & Warrick, A. W. Water distribution under trickle irrigation predicted using artificial neural networks. *J. Eng. Math.* **64**, 207–218 (2009).

[40] Lazarovitch, N., Šimůnek, J. & Shani, U. System-dependent boundary condition for water flow from subsurface source. *Soil Sci. Soc. Am. J.* **69**, 46–50 (2005).

[41] Rana, B. et al. Water budgeting in conservation agriculture-based sub-surface drip irrigation using HYDRUS-2D in rice under annual rotation with wheat in Western Indo-Gangetic Plains. *Field Crops Res.* **282**, 108519 (2022).

[42] Ekhmaj, A. I., Amin, M. S. M., Salim, S. & Zakaria, A. A. Wetted surface radius under point-source trickle irrigation in sandy soil. *Int. Agric. Eng. J.* **14**, 67–75 (2005).

[43] Ekhmaj, A. I., Amin, M. S. M., Salim, S. & Zakaria, A. Wetted surface radius under point-source trickle irrigation in sandy soil. *Int. Agric. Eng. J.* **14**, 67–75 (2005).

[44] Zur, B. Wetted soil volume as a design objective in trickle irrigation. *Irrig. Sci.* **16**, 101–105 (1996).

[45] Schwartzman, M. & Zur, B. Emitter spacing and geometry of wetted soil volume. *J. Irrig. Drain. Eng.* **112**, 242–253 (1986).

[46] Nafchi, R. F., Mosavi, F. & Parvanak, K. Experimental study of shape and volume of wetted soil in trickle irrigation method. *Afr. J. Agric. Res.* **6**, 458–466 (2011).

[47] Li, J., Zhang, J. & Li, B. Drip irrigation design based on wetted soil geometry and volume from a surface point source. 0300, (2013).

[48] Li, J., Zhang, J. & Li, B. Drip irrigation design based on wetted soil geometry and volume from a surface point source. In *2004 ASAE Annual Meeting 1American Society of Agricultural and Biological Engineers* (2004).

[49] Provenzano, G. & Using HYDRUS-2D simulation model to evaluate wetted soil volume in subsurface drip irrigation systems. *J. Irrig. Drain. Eng.* **133**, 342–349 (2007).

[50] Chen, J., Tan, Y. & Wu, Y. Analysis of infiltration of 2D trickle irrigation under multiple-line sources. *Hydrol. Process.* **22**, 2657–2666 (2008).

[51] Eugenio Coelho, F. & Or, D. Applicability of analytical solutions for flow from point sources to drip irrigation management. *Soil Sci. Soc. Am. J.* **61**, 1331–1341 (1997).

[52] Elmaloglou, S., Soulis, K. X. & Dercas, N. Simulation of soil water dynamics under surface drip irrigation from equidistant line sources. *Water Resour. Manag*. **27**, 4131–4148 (2013).

[53] Morianou, G., Karatzas, G. P., Arampatzis, G., Pisinaras, V. & Kourgialas, N. N. Assessing soil water dynamics in a drip-irrigated grapefruit orchard using the HYDRUS 2D/3D model: A comparison of unimodal and bimodal hydraulic functions. *Agronomy* **15**, 504 (2025).

[54] Elmaloglou, S. & Diamantopoulos, E. Simulation of soil water dynamics under subsurface drip irrigation from line sources. *Agric. Water Manag*. **96**, 1587–1595 (2009).

[55] Vital, S., Geza, M., Xu, S., Sexton, P. & Graham, C. Assessment and Hydrus (2D/3D) simulation of soil moisture dynamics under contrasting tillage management in rainfed cropping systems. *Vadose Zone J.* **24**, (2025).

[56] Šimůnek, J., Van Genuchten, M. T. & Šejna, M. The HYDRUS software package for simulating two-and three-dimensional movement of water, heat, and multiple solutes in variably-saturated media. *Technical Manual, Version* 1, 241 (2006).

[57] Simunek, J., Sejna, M. & Van Genuchten, M. T. *The HYDRUS-2D Software Package*. (International Ground Water Modeling Center, 1999).

[58] Šimůnek, J., Šejna, M. & Van Genuchten, M. T. *The HYDRUS-2D Software Package for Simulating the Two-Dimensional Movement of Water,Heat, and Multiple Solutes in Variably-Saturated Media: Version 2.0*. (US Salinity Laboratory, Agricultural Research Service, US Department of ..., 1999).

[59] Simunek, J., Van Genuchten, M. T. & Sejna, M. The HYDRUS-1D software package for simulating the one-dimensional movement of water, heat, and multiple solutes in variably-saturated medi. (2005).

[60] Richards, L. A. Capillary conduction of liquids through porous mediums. *Phys. (College Park Md)*. **1**, 318–333 (1931).

[61] Mualem, Y. A new model for predicting the hydraulic conductivity of unsaturated porous media. *Water Resour. Res.* **12**, 513–522 (1976).

[62] van Genuchten, M. Th. A closed-form equation for predicting the hydraulic conductivity of unsaturated soils. *Soil Sci. Soc. Am. J.* **44**, 892–898 (1980).

[63] Schaap, M. G., Leij, F. J., Van Genuchten, M. T. & Rosetta A computer program for estimating soil hydraulic parameters with hierarchical pedotransfer functions. *J. Hydrol. (Amst)*. **251**, 163–176 (2001).

[64] Li, J., Li, Y., Feng, C., Wen, M. & Zhang, Y. Multiscale modeling of hydraulic fracture propagation and design optimization in heterogeneous oil–gas reservoirs. *Geomech. Energy Environ.* **45**, 100806 (2026).

[65] Xu, K. et al. Analysis of dynamic coupling characteristics and multi-constraint optimization of a proton exchange membrane fuel cell considering membrane degradation. *Fuel* **404**, 136275 (2026).

[66] Zhao, J. et al. A fractional-order SSIM-based Gaussian loss with long-range memory for dense VSLAM. *Fractal Fract.* **9**, 744 (2025).

[67] Pan, D. et al. Synergistic optimization of nanosilica sol-calcium aluminate cement composite grout by silica micropowder: Effects on microstructure and sealing performance. *Case Stud. Constr. Mater.* **24**, e05894 (2026).

[68] Yang, Q. et al. Physical model test and numerical modeling of cross-sectional shape effect on evolution mechanism of time-delayed deformation and rockburst in deep tunnels. *Rock. Mech. Rock. Eng.* **59**, 2015–2043 (2026).

[69] Wu, Y. et al. Establishment of a Modelica model for flow metering valve controlled pump and analysis on delivery and leakage characteristics. *Energy* **339**, 139034 (2025).

[70] Lv, C., Ji, Z., Yang, T., Zhao, H. & Zhang, H. Numerical simulation of deformation and breakage of compound droplet in air flow. *Phys. Fluids* **36**, (2024).

[71] Ding, P. et al. Fractional derivative viscoelastic models based on L2-1σ formula: modelling and numerical application. *Comput. Geotech.* **189**, 107633 (2026).

[72] Willmott, C. J. On the validation of models. *Phys. Geogr.* **2**, 184–194 (1981).

[73] Willmott, C. J. & Matsuura, K. Advantages of the mean absolute error (MAE) over the root mean square error (RMSE) in assessing average model performance. *Clim. Res.* **30**, 79–82 (2005).

[74] Nash, J. E. & Sutcliffe, J. V. River flow forecasting through conceptual models part I — A discussion of principles. *J. Hydrol. (Amst)*. **10**, 282–290 (1970).

[75] Feddes, R. A., Bresler, E. & Neuman, S. P. Field test of a modified numerical model for water uptake by root systems. *Water Resour. Res.* **10**, 1199–1206 (1974).

[76] Abd El-Wahed, M. H. & Ali, E. A. Effect of irrigation systems, amounts of irrigation water and mulching on corn yield, water use efficiency and net profit. *Agric. Water Manag*. **120**, 64–71 (2013).

[77] Kumar, A., Sonkar, I. & Sarmah, R. Modeling root zone water and salt transport using matric flux potential based root water uptake distribution. *J. Hydrol. (Amst)*. **630**, 130712 (2024).

[78] Liang, S. et al. Agricultural practices influence phosphorus transport and ecosystem health in rice-paddy systems: Insights from HYDRUS-1D simulations. *Agric. Ecosyst. Environ.* **385**, 109581 (2025).

[79] Ashrafi, A., van Genuchten, M. T., Ghanbarian, B. & Ebrahimian, H. Analytical solute transport modeling of furrow fertigation using the STANMOD software package. *J. Hydrol. Hydromechan*. **73**, 200–209 (2025).

